# Effects of Embodied Learning and Digital Platform on the Retention of Physics Content: Centripetal Force

**DOI:** 10.3389/fpsyg.2016.01819

**Published:** 2016-11-25

**Authors:** Mina C. Johnson-Glenberg, Colleen Megowan-Romanowicz, David A. Birchfield, Caroline Savio-Ramos

**Affiliations:** ^1^Department of Psychology, Arizona State University, TempeAZ, USA; ^2^Behavioural Sciences Institute, Radboud UniversityNijmegen, Netherlands; ^3^American Modeling Teachers Association, SacramentoCA, USA; ^4^SMALLab Learning, LLC, North HollywoodCA, USA; ^5^Mary Lou Fulton Teachers College, Arizona State University, TempeAZ, USA

**Keywords:** embodied learning, STEM, education, mixed reality, virtual reality, centripetal force, design principles

## Abstract

Embodiment theory proposes that knowledge is grounded in sensorimotor systems, and that learning can be facilitated to the extent that lessons can be mapped to these systems. This study with 109 college-age participants addresses two overarching questions: (a) how are immediate and delayed learning gains affected by the degree to which a lesson is embodied, and (b) how do the affordances of three different educational platforms affect immediate and delayed learning? Six 50 min-long lessons on centripetal force were created. The first factor was the degree of embodiment with two levels: (1) low and (2) high. The second factor was platform with three levels: (1) a large scale “mixed reality” immersive environment containing both digital and hands-on components called *SMALLab*, (2) an interactive whiteboard system, and (3) a mouse-driven desktop computer. Pre-tests, post-tests, and 1-week follow-up (retention or delayed learning gains) tests were administered resulting in a 2 × 3 × 3 design. Two knowledge subtests were analyzed, one that relied on more declarative knowledge and one that relied on more generative knowledge, e.g., hand-drawing vectors. Regardless of condition, participants made significant immediate learning gains from pre-test to post-test. There were no significant main effects or interactions due to platform or embodiment on immediate learning. However, from post-test to follow-up the level of embodiment interacted significantly with time, such that participants in the high embodiment conditions performed better on the subtest devoted to generative knowledge questions. We posit that better retention of certain types of knowledge can be seen over time when more embodiment is present during the encoding phase. This sort of retention may not appear on more traditional factual/declarative tests. Educational technology designers should consider using more sensorimotor feedback and gestural congruency when designing and opportunities for instructor professional development need to be provided as well.

## Introduction

Embodiment theory proposes that knowledge is grounded in sensorimotor systems. An extension of that proposition would be that learning can be facilitated to the extent that lessons are created that map to and activate those systems. For this series on embodiment across the life span, we focus on learning in adolescence and early adulthood. A taxonomy on embodied is education is presented and a randomized controlled trial is included that assesses the effects of learning platform and amount of embodiment on the learning of physics content.

Physics concepts are an obvious choice for the study of embodied learning because physical interactions are part of experience from the moment the brain/body begins to experience the world. A thrown object moves through the air along a parabolic trajectory. An object swung in a circle, like a yo-yo spun overhead, must be pulled toward the center of the circle by the string to remain in curvilinear motion. However, the world is complex, and people will induce incorrect concepts. For example, although we feel a force in our arm and physical body when swinging a yo-yo, it is not as clear that the same force is acting on the yo-yo. If the string breaks and the yo-yo flies off, which path will the yo-yo follow, a straight or a curved one?

Naïve beliefs about what causes observed behaviors of real objects in motion can be thought of as primitive “mental models” or phenomenological primitives (p-prims; DiSessa, 1988, 2000; Redish, 1994; Johnson-Laird, 1998; Hestenes, 2006), and these are frequently at odds with the expert models of physicists (Sengupta and Wilensky, 2009). These pieces of intuitive knowledge about how the world works are powerful and preserved, even in the face of exceptions (DiSessa, 2000). Since a p-prim is considered self-explanatory it is rarely mentioned in an explanation, it remains implicit. It is this implicit knowledge structure into which new physics knowledge is assimilated by the novice.

Reiner et al. (2000) posit that it is our generalized knowledge of the properties of material substances and how they behave that is responsible for the collection of naïve beliefs that we draw upon when trying to learn a new physics concept. Our knowledge of substances includes the following properties: substances are ‘pushable,’ frictional, containable, consumable, locational, movable, stable, corpuscular (have surface area and volume), additive, inertial, and gravity-sensitive. Novice physics students tend to explain collisions as constrained by internal properties of moving objects such as a ball possessing a force (Halloun and Hestenes, 1985a), this would explain some misconceptions about objects in motion.

### The Choice of Centripetal Force

**FIGURE 1:**
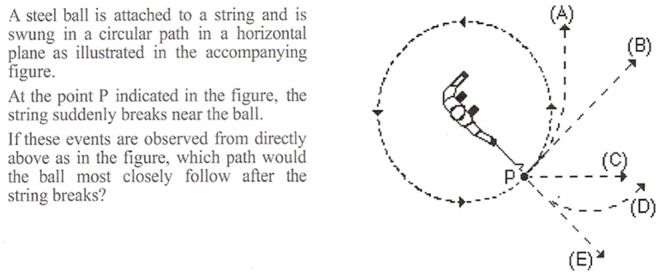
**Inspirational item from FCI test. (*Item 7 from the Force Concept Inventory, Hestenes, Wells, and Swackhamer, 1992-reprinted with permission from The American Modeling Teachers Association***.)

In the usual course of science, a research question is posed and then content and tests are created to answer the question. In an inspirational flip, the content of this multimedia study was actually inspired by a test item. The overarching goal was to assess how the amount of embodiment designed in to a lesson affected learning and the choice to focus on centripetal force (CF) arose from a classic item from the Force Concept Inventory (Hestenes et al., 1992). **Figure 1** shows the CF item that so readily lends itself to embodiment. Students often choose option A. When they pick a curved path after release, students are revealing an incorrect circular impetus notion of CF (McCloskey et al., 1980). We wanted to know whether a 50 min lesson on CF based on the principles of embodiment could help to overcome several misconceptions associated with CF, including the impetus model.

### Plain Folks Physics

Natural philosophers, physics education researchers, and learning scientists have devoted a great deal of effort to examining and describing how “just plain folks” understand physics concepts like force and motion (Viennot, 1979; McCloskey et al., 1980; Halloun and Hestenes, 1985a; DiSessa, 1988, 1993; Arons, 1997; Lakoff and Johnson, 1999). Surprisingly, the fact that we are experimental physicists since birth interferes with learning formal basic physics concepts. We bring to the learning experience well-entrenched ideas based on our observations and interactions with the real world, but those experiences do not easily allow us to separate effects of multiple forces (e.g., gravity, friction, and CFs) and can produce naïve conceptions that are often in conflict with formal physical laws (Halloun and Hestenes, 1985b; Lakoff and Johnson, 1999). McCloskey et al. (1980) described students’ intuitive physics knowledge as it relates to objects in circular motion, positing a sort of ‘circular impetus’ in that objects moving in a circle are endowed with an internal force that will keep them moving in a circular path. To address scientific misconceptions with new media and embodiment, we designed a study that varied along several physical and virtual dimensions. We chose fairly abstract content (CF) and focused on two common misconceptions: (a) that an object will continue in a circle when released from CF (impetus model), and (b) that the radius of distance effects the force in an additive monotonic manner, such that, the longer the yo-yo string, the greater the CF. This second misconception is sometimes called the “more is more” default. It may be based on Reif ’s (1995) suggestion that students’ reasoning in physics is based on retrieved plausible knowledge fragments, e.g., experiences or events, and the reasoning is tempered by the need for “cognitive efficiency.” That is, the expenditure of the least possible cognitive resources to arrive at a solution.

It is incontrovertible that physics can be difficult to learn. After a semester of college physics, the mean score for freshman physics majors at Harvard on an inventory of basic force concepts was a modest 77% (Hestenes et al., 1992). For many years, physics educators have merely presented formulae and proofs and assumed mastery would follow. For example, these instructors may believe that to learn about CF, a student need only be taught that F_C_ = mv^2^/r and the definitions of those terms to do well on traditional fill-in-the-blank tests. This is akin to believing the human information processing system is isomorphic to a computer and a modal. Yet, when students merely memorize and practice symbol manipulation they are still not achieving consistently high scores and we do not see generalization.

### Embodied Education

Suppose, however, that the human information processing system is a biological system that evolved in the service of action in the world (Wilson, 2003; Barsalou, 2008; Glenberg, 2008; Glenberg et al., 2013). In this case, learning may be more effective if it were based not so much on symbols and their manipulation, but on perceptual processes and the actions afforded in learning environments. In this case, learning about CF might be better facilitated by experiencing CF with the body. What if the variables in the equation above could be kinesthetically experienced? For example, what if learners could swing objects around their heads and feel the differences in the force when the mass of the object is altered, when the speed changes, and when the length of the tether is altered? Would that type of embodied learning result in a deeper knowledge structure? Would the new knowledge be retained longer? This new view of human information processing, that sensorimotor activation is also important, is the basis for the embodied cognition educational framework. This article assesses learning gains in three platforms and varies the amount to embodiment in each in an attempt to pull apart the most efficacious components. When we understand these components better, they can be folded into established classroom pedagogies from the learning sciences (e.g., situated learning) to create more powerful lessons.

The embodiment framework proposes that knowledge is highly dependent on sensorimotor activity. For education, when learners physiologically feel forces and exert agency over those forces during a lesson, they may more deeply comprehend forces in the world. Learning is primed by what we perceive, and what we expect in the world as we move about it, in addition to how we interact with the objects and situations discovered. In education, it may be that knowledge is not simply in “the extracted verbal or formal description of a situation, but rather in the perceptual interpretations and motoric interactions” in lessons (Goldstone et al., 2008). Such an approach would not seem to cover the learning of abstract materials in language, mathematics, or sciences. However, the notion of embodied simulation (Barsalou, 1999; Glenberg and Gallese, 2011) addresses this potential limitation. Learners may simulate mentally the constructs to be learned using perception and emotion as well.

The equation for CF, *F_c_ = mv^2^/r*, may at first appear to be a jumble of meaningless symbols. We propose that understanding the equation will be facilitated when learners map the symbols to sensorimotor experiences. Thus, a learner must map “*m*” to prior or new experiences with objects of differing mass and must coordinate experiences of speed (the *v^2^*), and radius (the *r*) to create a dynamic mental simulation of CF. The end product of this mental work is called ‘comprehension,’ it means that the learner has been able to create and apply appropriate mental simulations. Perceptual symbols may well underlie all cognition. But how are those first learned? We posit via interactions with the physical world. With this experiential basis established more complex and abstract thoughts can be created. One of our working theories is that reactivating some of the motor activities associated with a learned concept may aid in teaching new associated concepts. The hypothesis is that when the body uses meaningful (congruent) gestures and motoric actions to learn a new concept then the learning signal will be strengthened and primed to activate the knowledge upon recall. Our goal in this study was to create a lesson that would activate (or reactivate) many of the same sensorimotor and cognitive systems associated with CF while correcting several misconceptions.

#### Two Centripetal Force Misconceptions

A misconception that the majority of freshmen in physics still hold is that an object released from CF maintains a curvilinear trajectory after the point of release (Halloun and Hestenes, 1985b). This is called the impetus model. Second, students often come to the concept of CF with the misconception that a *longer* radius will always result *more* CF. The intervention was designed to give learners varying kinetic and visual experiences with different points of release and variable lengths of radius in a guided discovery manner in several mixed and augmented reality (AR) environments.

There are multiple non-mediated hands-on CF classroom experiences for students (Moore et al., 1981). A popular one involves a string, straw, and washers. Our lab believes that adding digital components to the hands-on experience can have powerful repercussions for learning. As Klahr et al. (2007) state, ... “inherent pragmatic advantages of virtual materials in science may make them the preferred instructional medium” (p. 183). Thus, many more trials can occur with a virtual mousetrap car compared to reconfiguring a new physical car for each hands-on trial. By adding digital components to hands-on components in a mixed or virtual reality (VR) platform, learners are able to easily pause during a lesson and reflect on what is happening in the instant. In our intervention learners were also able to observe the digital trail (called a ghost trail) left by the bob as it traveled after being released, thereby confirming whether the trajectory after release was straight or curved.

#### Gesture and Embodiment

Goldin-Meadow et al. (2001) propose that gesture and speech form an integrated, synergistic system in which effort expended in one modality can “lighten the load on the system as a whole,” that is, gesturing may actually shift some of the load from verbal working memory to other cognitive systems. Much of the earlier work on gesture and embodiment was done with videotaping experiments followed by human coding, but with affordable motion capture becoming more ubiquitous, more research is being done on grosser body movements and learning. Gestures (what might also be labeled as “instrumented gestures”) and full-body movements can be designed now to drive simulations. Digital simulations can be powerful learning aids when created with proper design heuristics (Mayer and Moreno, 2003). Especially larger display simulations can be engaging for leaners. Higher levels of engagement, and more positive attitudes toward science can be seen when whole body movement is integrated into large digitized science lessons (Lindgren et al., 2016). The medical education field has an ongoing history of researching the efficacy of simulations, immersive learning, and skill acquisition, as an example, surgeons perform better after training with the gesture-based Wii (Giannotti et al., 2013). In the next section we describe some differences between augmented and mixed reality (MR) platforms so the reader understands why certain design choices were made.

### Augmented and Mixed Reality

If using the body aids in learning, and receiving immediate digital feedback on actions can facilitate learning and skill acquisition, then perhaps we should be designing for platforms that integrate the two: gesture and user-driven simulation. Platforms that mesh virtual (digital worlds) with physical (kinetic worlds) are called MR environments. Impressive early taxonomic work on MR was done by Milgram and Kishino (1994). They proposed a three dimensional taxonomy and a “virtuality continuum” (Milgram and Kishino, 1994). The real world was one end point and a fully virtual world another end point on the continuum. In the middle was a “MR” world where physical and digital components would merge. It has become acceptable to use the term “AR” to also describe a segment of the middle space. We reserve the term “VR” to refer to fully enclosed, immersive spaces [e.g., head mounted displays (HMD) or four walled CAVEs].

Educational designers need guidance on how to design with gesture for embodied learning in AR/VR environments. Lindgren and Johnson-Glenberg (2013) recently published six precepts to follow while designing for embodied education and Johnson-Glenberg et al. (2014a) present an early version of an embodied taxonomy for education. In this article, the taxonomy is enhanced and includes a physics lesson and study done in *SMALLab* (Situated Multimedia Arts Learning Lab). *SMALLab* is a MR platform where learners hold trackable objects and can control and manipulate interactive digitized media. It is only very recently that guidelines for creating educational content for AR/VR and MR environments are starting to come out (Johnson-Glenberg, 2012; Wu et al., 2013). Thus, this is an emerging area.

### The Taxonomy for Educational Embodiment

According to our proposed taxonomy, for content to be considered minimally embodied it should contain three constructs: (a) sensorimotoric engagement, (b) gestural congruency, that is, how well-mapped the evoked gesture is to the content to be learned, and (c) evoke a sense of immersion. If content is an animation on small screen or monitor offering no interactivity to the user, then it should be called a “simulation” and not be referred to with the terms “embodied, virtual, mixed, or augmented reality.” The three axes or constructs were chosen to account for how the body might move and how “present” the learner might feel in the lesson. The taxonomy should be considered a work in progress. The three constructs of embodiment in education occur on three continuous axes, but are partitioned binarily as low and high in order to make it more tractable. The resulting eight sets are then binned along the one dimension of embodiment into four degrees. See **Table 1**. The 4th degree is the highest because the three constructs are highly present in the content; the 1st degree is the lowest because all constructs are low in the content. The 3rd and 2nd degrees each contain three sets with combinatorial mixtures of lows and highs. The edges between adjacent degrees can be considered fuzzy, but discernable differences are noticeable between degrees separated by more than one step (i.e., 4th and 2nd degree, or 1st and 3rd degree). In mathematics this ordering is called a “weak ordering” because the highest class (first set termed the 4th degree) has no predecessor, and the last class (last set termed 1st degree) has no successor. We note that it would be odd to purposefully design content that is high on sensorimotor input but low on gestural congruency (e.g., Why make a user swipe a finger back and forth across an entire tablet three times to turn one virtual page?).

**Table 1:** Construct magnitude within degrees in the Embodied Education Taxonomy; H, High, L, Low.

Degree	4th	3rd	3rd	3rd	2nd	2nd	2nd	1st
Embodiment construct								
Sensorimotor	H	H	H^∗^	L	L	L	H^∗^	L
Gestural congruency	H	H	L^∗^	H	L	H	L^∗^	L
Immersion	H	L	H	H	H	L	L	L

*^∗^It would be odd to require a large movement that was poorly mapped to the content to be learned.*

The degrees are:

4th degree = (a) Sensorimotor engagement – High. The system can map (via motion capture, etc.) the whole body, or any part of the body, which can act as the controller of the system. If locomotion is included then visual parallax is also engaged (Campos et al., 2000) and this further increases sensorimotor activation. (b) Gestural congruency – High. There are multiple instances of gestures that drive the system, and these are consistently designed to map to the content being learned. E.g., spinning the arm makes a virtual gear spin the same speed and direction on the screen. (c) Sense of immersion– High. A very large display is used so the learner perceives environment as very immersive, or a HMD can be used that covers a very large percentage of the field of vision (FOV); borders are not readily apparent (e.g., a participant might stand in the middle of a floor projection with a 21 foot diagonal and when looking down the boarders are not apparent). Participant appears to be fully engaged and/or reports feeling “in the world.”

3rd degree = (a) Sensorimotor engagement – The whole body could be used as the controller, but the user remains in one place [e.g., standing at an Interactive Whiteboard (IWB)]. (b) Gestural congruency – The system should contain one or more instances of this. (c) Sense of immersion – A large screen display or floor projection should induce the learner to perceive the environment as immersive; however, borders are usually present in the peripheral. A participant may report they on occasion felt they were “in the world.”

2nd degree = (a) Sensorimotor engagement – Learner is generally seated, but there is some upper body movement of the arm or fingers. (b) Gestural congruency – Probably not a defining feature of the content, although there is always some interactivity (e.g., finger swipe to advance, spin mouse for a circle on screen), (c) Sense of immersion – The display covers less than 50% of FOV and borders are always present no matter the fixation point (e.g., a 16 inch monitor, or tablet-sized screen).

1st degree = (a) Sensorimotor engagement – Low. Learner is generally seated, but there is some upper body movement, usually for a key press occurs. The learner is primarily *observing* a video/simulation. (b) Gestural congruency – Low. There is no learning-related mapping between gesture and content, the users’ movements are elicited primarily for navigation (e.g., tap for next screen). (c) Sense of immersion – Low. The display covers far less than 50% of FOV and borders are always present (e.g., small form display generally tablet or smartphone screen).

#### More on the three constructs

In this section the three constructs of embodiment in education are further explicated.

##### Sensorimotor engagement

More muscular movement engages more sensorimotor systems and this translates to larger areas in the sensorimotor cortex being activated. We propose that using larger learning gestures (e.g., moving an arm, rather than a finger) may result in a stronger learning signal. That is, using the arm with the shoulder joint as the fulcrum to simulate a water pump handle may be more meaningful in terms of learning than using the index finger. The reader may ask whether this sort of distinction really matters for the adult brain. Perhaps for a child it may be important to map the meaning of words to actions, but in the adult brain, packed with overlearned words, how much importance would the body metaphor hold for learning new concepts? Brain imaging studies reveal intriguing results. Hauk et al. (2004) measured brain activity using functional magnetic resonance imaging (fMRI) while people listened to action verbs such as *lick, pick*, and *kick*; significantly more somatotopic activation of the premotor and motor cortical systems that specifically control the mouth, the hands, and the legs (respectively) was observed. If these overlearned words still activate specific motor areas in the adult brain, and if Goldin-Meadow et al.’s (2001) postulation is correct that gesturing helps to off-load cognition, then perhaps it makes sense to teach science content through the body. A compelling example comes from Kontra’s lab (Kontra et al., 2015), students who physically held two bicycle wheels spinning on an axle learned more about angular momentum compared to students who observed a partner holding the wheels. In an extension of their lab study, Kontra et al. (2015) pushed further with an fMRI experiment that revealed that the action group did better than the observe group on tests, and that the level of the BOLD signal in the brain motor regions of interest (left M1/S1) *significantly predicted* test performance (*r* = 0.58, *p* = < 0.009) for both groups. Kontra et al. (2015) tout this as a model that explains how physical experience, relative to observation, increases “activation of the sensorimotor systems important for representing dynamic physical concepts.” (p. 6).

Surely emotion and the other senses play a large role in learning and have a place in an embodied theory for education. This article is primarily focused on gesture and kinetics. Nonetheless, it should be mentioned that emotions, and all of the senses, including proprioception, have a place in embodiment theory. If the learning platform and the content afford strong reasons for using more modalities, then those should be considered during design.

##### Gestural congruency

Segal et al. (2010) show that when students interacted in a more gesturally congruent manner (i.e., tapping out a number versus selecting a symbol for the number) on a tablet-based math game, the tapping students made fewer errors on a post-test. Koch et al. (2011) report that participants reacted faster in the condition that meshed Stroop choices with congruent gestures compared to those in the incongruent gesture group. Our lab’s recent studies also support the superiority of gestural congruency for learning about vectors (Johnson-Glenberg et al., submitted).

These gestures are based on what Antle and others call “body metaphors.” Antle designed her Sound Maker virtual reality system (Antle et al., 2009) using viable physical mappings for volume, tempo, pitch, and rhythm, e.g., tempo was associated with speed of movement through the room, pitch was associated with movement up and down in 3D space, or toward and away from in a 2D space, etc. She counsels for “interactional mappings that preserve structural isomorphisms between lived experience and the target domain.” Thus, designers should strive for gestural congruency using movements that coincide with real life and cultural experiences, e.g., raising the hand upward usually signifies something going higher. There is a history of this research in cognitive psychology using other names like Self-Performed Tasks (SPT; Engelkamp and Zimmer, 1994; Engelkamp, 2001). A representative study from Engelkamp would compare three groups of participants: one that heard a list of unrelated action phrases (“lift the hat”), one that performed the action without the object, and one that performed the task with the object. The consistent finding was that the self-performing participants recalled more of the phrases than those who merely heard the phrases. When assessing for learning after actions have been performed we cite the encoding specificity hypothesis (Tulving and Tomson, 1973) which holds that content will be better recalled when the cues match the method with which the content was encoded. In our modern and new world of digitization, we are able to easily add the virtual objects to any lesson, what are the effects of that?

##### Immersion

Coomans and Timmermans (1997) state that immersion is “...the feeling of being deeply engaged... (in) a make believe world as if it was real.” (p. 279). Immersion is subjective, difficult to precisely quantify, and results have been mixed on its effects on learning. In the medical research community the concept of immersion in a task is well-received. A meta-analysis by Miller and Bugnariu (2016) showed that for high-immersion virtual environments treatment response was overwhelmingly positive for those with autism spectrum disorder who were learning social skills. Gutiérrez et al. (2007) showed that students who learned how to treat a head trauma victim via a proprietary virtual HMD (they called this the “full immersion” condition) showed significantly better learning than students who learned on a laptop monitor (called “partial immersion” condition). In the paper it is not explained why those condition definitions were chosen. However, in a small *n* correlational study run by Bailenson’s group (Bailey et al., 2012), participants learned multiple environmental messages while in a VR shower. They then filled out a five item Physical Presence questionnaire and the higher the presence score, the significantly less content participants remembered on a cued recall task. This negative correlation had not been predicted. The authors speculate that after a highly vivid sensory experience, participants may have had limited cognitive resources left over to dedicate to the memory task. Thus, the idea that an immersive VR or MR environment will indiscriminately enhance learning has not been fully supported yet.

The field needs to rigorously define and operationalize immersion for the sake of learning, then we will be better able to weave instances of congruent gestures into effective and immersive educational environments. Slater and Wilbur (1997) proposed that the immersive capability of a virtual environment depends on the degree to which it is “inclusive, extensive, surrounding, vivid, and matching.” Each of these components influences, but is not the sole determinant of the user’s perceptual experience. *Inclusive* refers to whether signals pertaining to the physical world have been eliminated (e.g., joystick, weight of wearables, etc.). *Extensive* refers to the number of sensory modalities that are part of the experience. *Surrounding* refers to the visual presentation including FOV and the degree to which the physical world is shut out. *Vivid* refers to the fidelity and resolution of the simulation. *Matching* refers to whether the viewpoint of the environment is modified to match the user’s perspective (e.g., in an HMD when the user moves left, the environment moves as well). The construct of immersion is complex with several measurable components, under the category of inclusive we should also place physiomarkers (i.e., heart rate, skin conductance, pupil dilation, etc). For our study, we focus primarily on the sense of *surrounding* for immersion. Thus, FOV served as our primary marker or determinant. Borders defined the edge of the learning platform and three very distinct FOV’s existed in each platform, with *SMALLab*, the MR platform to be described in the next section, being the most immersive and extensive.

### Crossing Learning Platform with Amount of Embodiment

The study was designed to use a mixture of platforms that are readily available in schools and at least one that is innovative and very immersive. The *SMALLab* platform provided the greatest opportunity for use of the whole body and locomotion (and consequently, the greatest range of sensorimotor experiences). These platforms are sometimes called EMRELEs for Embodied Mixed Reality Learning Environments and one might expect the greatest learning gains to be seen in the condition with more whole body activity (Johnson-Glenberg et al., 2014a). To ask the question of how learning gains are affected by platform crossed with embodiment, three different platforms were selected: *SMALLab* with a very large projected floor display [21 foot (252 inch) diagonal], an IWB (78 inch diagonal), and a traditional desktop with a monitor (16 inch diagonal). The hypothesis is that the high embodied, 4th degree *SMALLab* platform which encourages larger, stronger sensorimotor signals (by actually spinning objects), and a greater sense of immersion (defined by FOV) will result in better learning and greater delayed learning gains. If stronger memory traces can be practiced with more haptic and large-display visual feedback, then perhaps these traces will aid in bootstrapping new knowledge to the learner’s existing knowledge structures. Six conditions that varied in amount of embodiment (low versus high) as afforded by the three platforms were created.

#### Delayed Learning Gains

The final factor in the experiment was time. As the work on memory consolidation continues (Walker and Stickgold, 2004; Stickgold and Walker, 2007, 2013), it seems critical to ask whether there were group differential effects on knowledge over time. A student may not be able to answer some questions based on more labile forms of memory, for example, a memorized verbal description of a rule or definition after a delay. However, if adding a strong sensorimotor trace increases memory via more complex connections, then a different slope (interaction) for content memory by condition may emerge. With time, group differences might emerge.

In sum, the primary question was whether levels of embodiment in mediated learning platforms affect immediate and delayed learning. We hypothesized that a congruency between learned content and assessment metric might be felicitous for those who learned in a more isomorphic manner, that is, those who used grosser gestures to and more sensorimotor engagement might perform better on a test that included movement via drawing.

Three hypotheses were tested and our predictions and rationales are listed after each.

(1) Platform should be predictive of both immediate gains and delayed learning gains. We predict that the platforms that afford more embodiment and haptic interaction will show greater comparative gains. That is, learners using larger gestures on a platform like an IWB should learn more than those on a computer using more constrained mouse-driven movements; however, the greatest gains should be seen on the MR platform called *SMALLab* because it affords the most sensorimotor engagement as well as locomotion.
(2) Level of embodiment should be positively predictive of both immediate and delayed learning gains. This is hypothesized because a high degree of embodiment implies that the gestures used while encoding were congruent to the content to be learned. This congruency should strengthen the memory trace and facilitate recall of the newly learned content for both post-tests and delayed tests. The greater the amount of embodied gestures that are congruently mapped to the content, then the better the participants should perform on all post-intervention assessments.
(3) The *SMALLab* high embodied condition should be the one to demonstrate the greatest learning gains. It is the platform that affords the greatest amount of embodiment. If learners are encoding in a more embodied and immersive manner, they may be better at over-riding incorrect analogies about how spinning objects operate in the real world. We want to encourage novice learners’ unstable and incorrect mental models to begin to resemble the experts’ more veridical models. By using larger body gestures in immersive environments learners may be better able to rehearse and “cohere their pieces” of knowledge, or p-prims, into the correct structure for understanding complex physics concepts.

## Materials and Methods

### Participants

From the introductory psychology research pool at a large university, 110 participants were recruited. This study was carried out under the auspices of the Internal Review Board at Arizona State University and with written informed consent from all participants. Because some computer-collected data were lost, some analyses have data from only 105 participants. In addition, data from one participant were eliminated because the participant’s score on the post-test was more than four standard deviations below the mean. Of the remaining 109 participants, 32% were female, 85% were native English speakers, the median number of high school physics courses completed was 1.0 (with a reported range of 0–4 or more), and the median number of college physics courses was 0 (range of 0–3).

### Design

Before starting to code the CF content, we held over a dozen design meetings focused on how to create the conditions. It should be stated again that the constructs in the taxonomy are not orthogonal: sensorimotor activation, gestural congruency, and immersion. In addition, we did not have the resources to run every variation, i.e., each construct varied by high versus low, by the three platforms. Indeed, as noted in **Table 1**, some conditions are not particularly ecologically valid for educational purposes (why create a non-sensical high sensorimotor/no gestural congruency condition, i.e., doing jumping jacks to learn about CF?). Some educational platforms afford various immutable properties. To simply turn off “immersion” in *SMALLab* is non-felicitous because *SMALLab* is at its core a large projection, motion capture environment. Turning off the graphics with real-time feedback would have rendered it a human-tutored lesson with a spinning manipulable; that platform would not have addressed timely new media and design issues.

Working within the stated framework, we assumed low versus high embodiment (as conceptualized by the three constructs simultaneously) and crossed the two levels of embodiment with the three platforms (i.e., *SMALLab*, IWB, and desktop), this resulted in six conditions. We crossed platform with embodiment and not immersion because high-immersion is folded into the definition of high embodiment. Second, we are asking questions relevant to the current state of educational technology. The types of platforms (and their affordances) that could be placed in modern classrooms drove the study. At the time of the study only desktops and IWB’s were in general use. There were few tablets and it would not be expeditious to research non-existent platforms (i.e., *SMALLab* without a floor projection).

The experiment was a mixed 2 × 3 × 3 factorial design. The first two factors were manipulated between-subjects: (1) embodiment with two levels – low or high, and 2) platform with three levels – Desktop, IWB, or SMALLab. **Table 2** gives examples of the salient differences between the six conditions. The within-subjects factor, test time, had three levels: pre-test (same day), post-test (immediately after instruction- same day), and follow-up (an average of 1 week after instruction).

**Table 2:** Salient differences between conditions.

		Desktop	Interactive whiteboard	*SMALLab*
**Platform**				
Physical description	Control device	Mouse	Trackable pen	Trackable manipulables
	Body position	Seated	Standing	Standing, spinning
	Display size	16 inch diag.	78 inch diag.	252 inch diag.
**Embodiment conditions**				
Low embodiment	Sensorimotor	Hand moves mouse to control virtual bob	Hand and arm hold pen to control virtual bob	Hand and arm hold trackable wand to control virtual bob
	Gestural Congruency	Mouse controls speed slider on screen, left to right	Tracking pen controls speed slider on screen left to right	Hand controls speed slider projected on floor, left or right
	Immersiveness-primarily FOV	Low	Medium	High
High embodiment	Sensorimotor	Hand moves mouse to control virtual bob	Hand and arm hold pen to control virtual bob	Hand and arm hold physical manipulables to control a physical bob, and body spins in circle
	Gestural Congruency	Mouse moves in small circles, maps to circular movement of virtual bob	Pen moves in large circles, maps to circular movement of virtual bob	Swinging the physical bob overhead. Also, the whole body spins around to release the bob
	Immersiveness-primarily FOV	Low	Medium	High

*Except for key device nouns the experimenter script did not vary between conditions.*

### Materials and Procedure

All conditions included a pre-test, watching a 3-min vocabulary animated video (wherein the four CF terms in the equation were explained), the instructional intervention (i.e., experimental manipulation), a post-test, and a delayed follow-up test. The instructional intervention – hereafter referred to as the lesson – consisted of the participant interacting one-on-one with one of two experimenters to learn key concepts and the proportional relationships represented in the CF equation. Participants manipulated either a real or a simulated “bob” that spun around a fixed point.

### The Three Platforms and Lesson Variations

A group of five high school physics teachers met for multiple sessions to co-design the content. A master script was written for the two experimenters to follow as they instructed participants. The first coded version of the lesson was for the extreme lesson, i.e., the “all bells and whistles” version, that was deemed to be 4th degree with the most embodiment. The first lesson was high on all constructs: sensorimotor, gestural congruency, and immersion and that lesson was best afforded by the platform called *SMALLab*. The following lessons were then tailored down along the dimensions of gross body movements, congruency of gesture, and immersion as described in the taxonomy, until ending up with the desktop and mouse version with little interactivity. It is important to note that the instructions and question prompts from the experimenters remained the same throughout the six conditions, only short phrases were altered “spin the mouse” versus “sin the bob.” All lessons were equated for time on task.

In the extreme lesson, i.e., high embodied *SMALLab*, the three variables in the CF equation were instantiated in a multimodal manner. For example, (1) *radius* corresponded directly to the length of a physical string attached to a bob that was spun overhead, (2) *mass* corresponded directly to number of weight packets placed inside the spinning bob, and (3) *velocity* was controlled directly by the participant spinning the tangible bob overhead with his/her hand. Thus, the participant received real-time sensorimotor/haptic feedback, as well as aural and virtual visual feedback (e.g., increase in pitch as the bob swung faster, bar charts corresponded to bob’s speed). The pitch increase (aural) and bar charts (visual) feedback were given in all six lessons.

In the low embodied condition the participants had control over the bob, but in an indirect manner – through a virtual slider. The media-rich dynamic graphics in the animations were visually engaging and as the speed of the spinning bob increased pitch always increased accordingly. Thus, we make the distinction between high and low embodiment, and we avoid the descriptor of “no embodiment.” One of the greatest differences between the low and high conditions that might not be readily apparent in **Table 1** is that controlling the bob was not “well mapped” in the low embodied conditions. Participants in the low embodied *SMALLab* condition used a tracking wand to move a slider to increase speed of the bob. Whereas, in the high embodied conditions, the physical act of spinning the mouse or pen, or swinging the bob overhead actually *corresponded directly to and drove* the speed of the virtual bob. In juxtaposition, in the low embodied conditions, the speed of the bob was driven by lateral placement on a virtual slider. Thus, location of a point along a short horizontal slider drove speed and not a congruent action. There was some agency involved in selecting that speed, but there was not a one-to-one, direct gesture-driven mapping associated with the speed of the bob. This is what is meant by “not well mapped.” To see a video to help conceptualize, please visit www.embodied-games.com/games/all/centripetal-force or https://www.youtube.com/watch?v=oFiXtcXRpVE.

#### Condition 1 – High Embodied *SMALLab*

**FIGURE 2:**
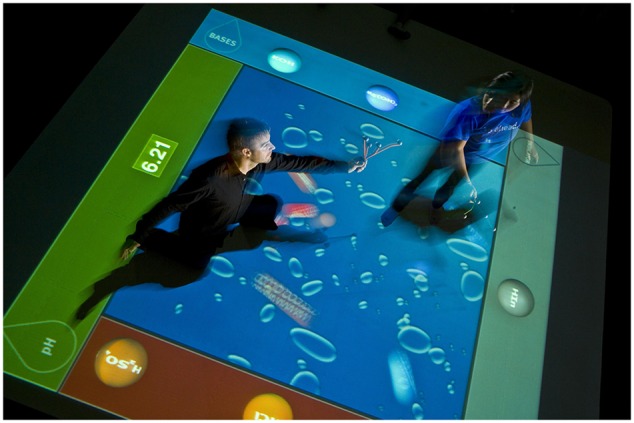
**Example of *SMALLab* floor projection with marker-based motion capture**.

The Situated Multimedia Arts Learning Lab (*SMALLab*) is a 15 × 15 × 15 feet interactive space designed to engage multiple sensory systems including vision, audition, and kinesthetics. See **Figure 2** for an example of the how the floor projection works.

The MR environment includes both digital components (projected graphics on the floor) and tangible, physical components (motion tracking wands and manipulable handheld objects). The platform provides an engaging mesh of the real and the virtual. The system uses 12 infrared NaturalPoint *Optitrack* cameras for real-time motion tracking of handheld rigid-body objects. Participants manipulate projected images on the floor with the handhelds. The wands and tracked spinning bobs are tracked in *X, Y, Z* coordinates with millimeter precision. One of the notable differences between this platform and a traditional desktop or IWB platform is that participants can locomote through the immersive environment thus providing multiple opportunities for congruency between dimensions of action and dimensions of the content being learned, as well as parallax (Johnson-Glenberg et al., 2014a) and Campos (Campos et al., 2000) for reasons why locomotion may represent a special case of embodied learning from a developmental perspective.

##### New tangibles

**FIGURE 3:**
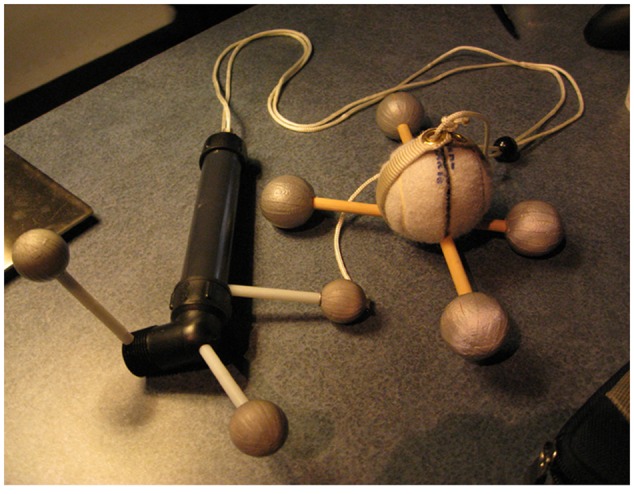
**The “Swinger” used in the high embodied *SMALLab* condition**.

Two new tangible objects were constructed for this study – a ‘swinger’ and a ‘flinger.’ **Figure 3** shows the swinger, it is a tracked bob or ball on a string of varying length. The mass inside the ball can be varied during the lesson by inserting taped weight packets inside the bob, and the radius can be adjusted by switching between two lengths of string.

**FIGURE 4:**
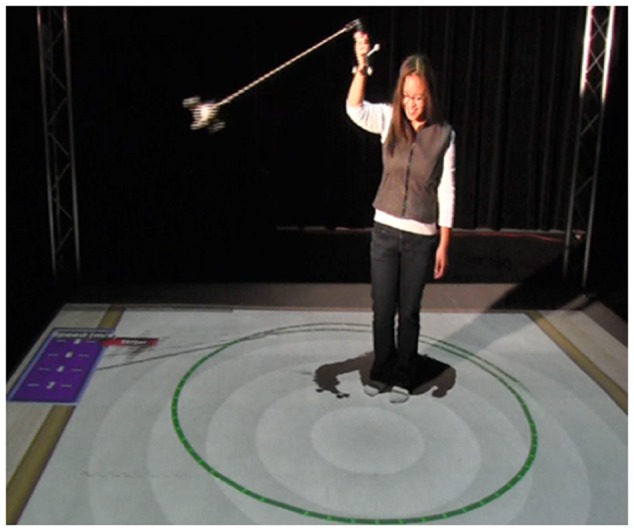
**The “Swinger” in action. Note dynamic simulations projected on the floor giving real-time feedback**.

**Figure 4** shows a student using the swinger in the high embodiment condition. The adjustable string was created to address the radius misconception that more is more. The swinger was always swung overhead.

**FIGURE 5:**
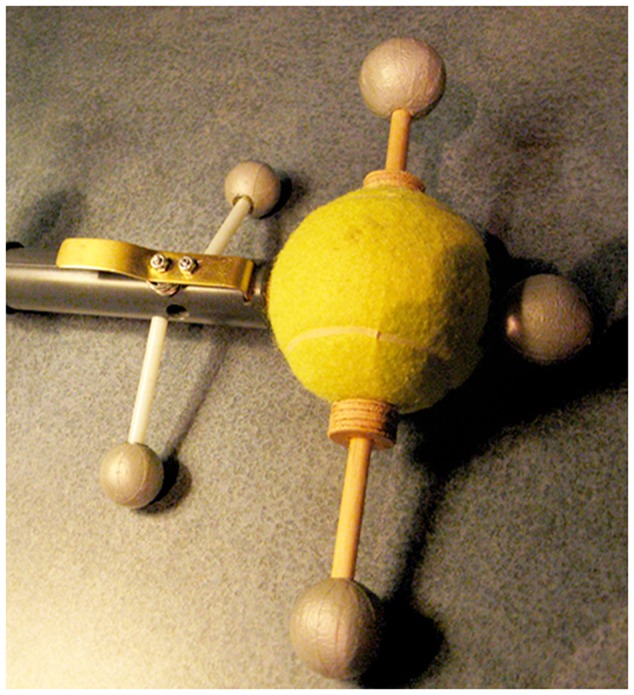
**Close up of the “Flinger” tangible**. The brass lever releases the bob; both the handheld component and bob are tracked.

The second tangible was the flinger. See **Figure 5**. This dual-component wand was used to assess the learning of trajectory at point of release. The brass lever on the top of the handle serves as the release mechanism. Participants physically spun or rotated their entire bodies around holding the flinger in front so they could watch it at all times. They then released the bob at a time of their choosing to hit a target on the floor. That is, the tennis ball (the ‘bob’ part) of the unit would disengage and fly from the tracked handle when the brass lever was depressed. The smaller tracked spheres on both components are covered with retro-reflective tape and this allows the IR cameras to map the positions of both the handle and the moving bob. Thus, when the bob is released, both the handle in the hand and the released bob moving through the air can be tracked for several seconds. Participants are able to observe how the bob flies at the point of release and begin to address the impetus misconception regarding trajectory at point of release.

**FIGURE 6:**
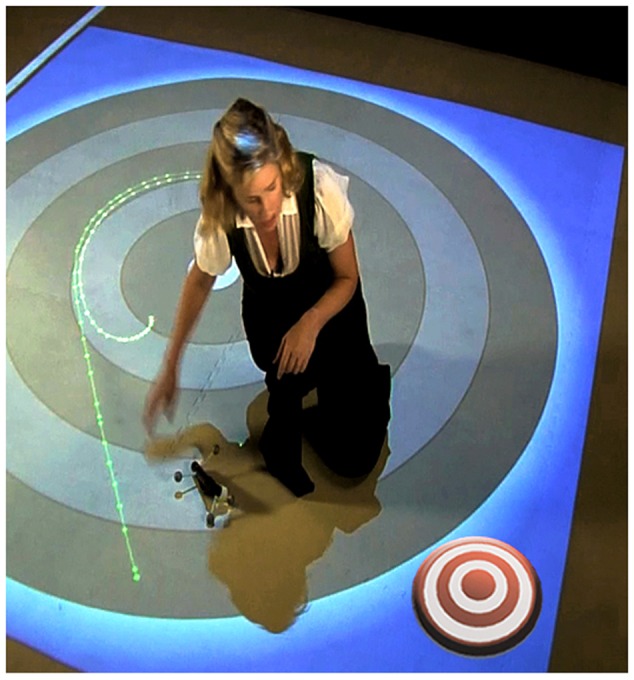
**The Target Game.** There are two “ghost trails” on the floor. The line that continues to curve represents the handheld component. The line that straightens represents the bob’s tangent at point of release.

**Figure 6** shows a user who has released the bob trying to hit the virtual bulls-eye target on the ground during the Target Game. It also shows the mediated ghost trail feedback on the floor projection. It is important that both sets of tangible objects are tracked. Again, users receive visual feedback on placement of both the handle (in **Figure 6** the curling line) and the traveling bob (the straight line) from user-created movements. The visual feedback provides irrefutable evidence that the bob travels in a straight line after release.

Given the full-body kinesthetic experience in *SMALLab*, this lesson is considered highly embodied. The experimenters followed a memorized script. The script did not vary substantively between conditions, only a noun or two might change. Experimenter effects were controlled for in this manner. Each element in the CF equation was introduced one at a time using a “guided discovery” method so that participants would not be overwhelmed with the physics concepts.

#### Condition 2 – Low Embodied *SMALLab*

In the low embodied *SMALLab* condition, participants used a different rigid-body trackable object. This was an extant 3D-printed plastic wand that has been used for other studies. It has a unique configuration of retro-reflective spheres and performs much like a “wireless mouse.” Participants in this condition used the plastic wand to control the virtual slider and signal a release from spin via *X, Y, Z* placement of the wand.

**FIGURE 7:**
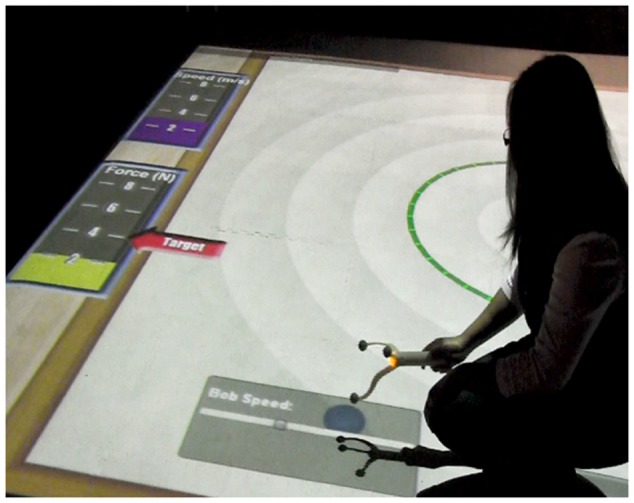
**Student in low embodied condition using trackable wand to change Speed.** (example of low gestural congruency).

In all three low embodied conditions, the participant controlled speed of spin with a horizontal virtual slider. In this *SMALLab* low embodied condition the slider graphic can be seen in the bottom of **Figure 7**. Again, the participants had a small amount of agency over the speed of the bob, but their gestures or actions were not highly congruent (as in the previous condition). The digital slider action is left-to-right linear and does not afford the physical, circular kinesthetics of swinging an object overhead. In addition, speed of moving the wand did not correspond to speed of the bob spinning, only placement of a virtual marker on the slider altered speed.

**Figure 7** shows a student adjusting the simulated bob speed using the wand over the slider. Note the bar graph on the upper left that shows immediate feedback as the participant attempts to match a target speed, similar to the high embodied condition. When the participant wished to release the bob from its virtually tethered spin and hit the target during the Target Game, s/he merely raised the wand up (in the *Z* axis) and the virtual bob was released. The virtual bob left the same type of ghost trail on the floor as the physical bob.

#### Condition 3 – High Embodied Interactive Whiteboard

In the IWB platform, the participants used the tracking pen provided with the Promethean *ACTIVboard*. In the high embodied condition, participants started the bob spinning by directly moving the tracking pen in a circular motion on the large vertical board. The velocity of the hand movement directly controlled the velocity of the bob, i.e., the virtual bob is linked to the pen’s tip. This would be considered highly gesturally congruent. For the Target Game or “trajectory at release” phase of the lesson, participants lifted their index finger from a trigger-button on the pen to release the bob.

**FIGURE 8:**
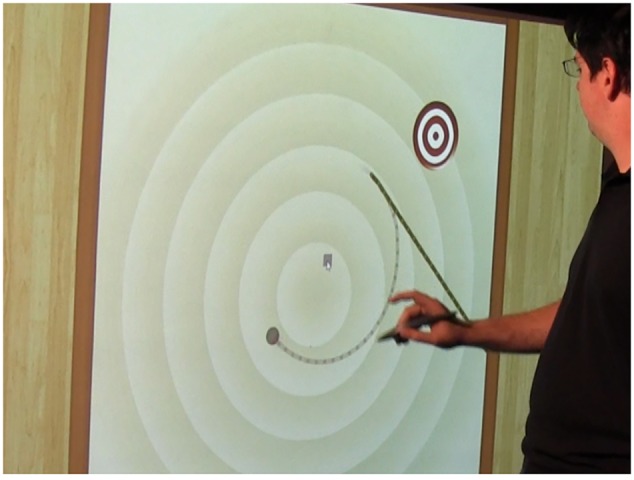
**Participant at the whiteboard in the high embodied Interactive Whiteboard (IWB) condition using the tracking pen**.

**Figure 8** shows a student who missed hitting the target on his first try. It should be noted that in all six conditions during the Target Game the bob’s path was always tracked and presented via a ghost trail.

#### Condition 4 – Low Embodied IWB

In the low embodied condition on the IWB participants again interacted with the left-to-right virtual slider at the bottom of the IWB screen (similar to the gesture used in all other low embodied conditions). Moving the pen along the slider increased or decreased the spin, but not in a direct one to one manner, what mattered was placement of a marker on the horizontal slider.

To release the bob from the spin during the Target Game in the low embodied condition, the student watched the bob spin and then tapped a virtual “release” button on the IWB screen at the desired moment of release. Thus, there was some agency associated with the task, but it would still be considered to have low levels of gestural congruency. In all three low embodied conditions for the Target Game, participants were always able to choose the release points.

#### Condition 5 – High Embodied Desktop

In the desktop platform, the participants viewed a 16 inch monitor and used a Windows 7 tower machine on the floor. Participants sat at a desk and used the mouse on a table to start the bob spinning in a circular motion. The direction and velocity of the hand controlled and directly mapped to the direction and velocity of the virtual bob, so there was gesturally congruent feedback. For trajectory at release during the Target Game, the participant lifted the index finger off the left mouse button to release the bob.

#### Condition 6 – Low Embodied Desktop

In the low embodied condition, participants were presented with the same virtual left to right slider as was developed for the two other low embodied conditions. They used the mouse to click and drag the marker on the virtual slider to affect bob speed. In all low AND high embodied conditions participants generated their own real-time data for the spinning sections. It should be noted, in all low embodied conditions they did not merely watch animations, but had varying degrees of agency over all simulations. Participants saw graphs and arrows and feedback related to velocities that were self-generated, albeit in a low embodied manner via the slider. For the Target Game participants clicked on an on screen button to control the moment of the bob’s release.

### Assessment Measures

A 20 item test was designed in conjunction with two high school physics teachers. See Appendix A. All three tests were invariant, except that the post-test and follow-up tests included one final item (item 21 an open-ended question) that was not used in these analyses as it was not included in the pre-test. Two distinct subtests were designed, a computerized declarative subtest and a hand-drawn generative subtest.

#### Declarative Knowledge Computerized Subtest

The first, on-line subtest (items 1–13) was administered using a computer. It was designed to primarily tap memory for information explicitly presented during the lessons. The first five items in this section were open-ended questions querying definitions of CF terms. The definitions were scored on a 0–2 scale. Items 6–13 described or showed images of circular motion events and answer choices were displayed in a four item multiple choice format. Of these eight image-based items, four of them ended with the prompt, “Explain why you chose that answer.” Participants typed in responses and these were scored on a scale of 0–2, often these resulted in one word responses.

#### Generative Knowledge Subtest

**FIGURE 9:**
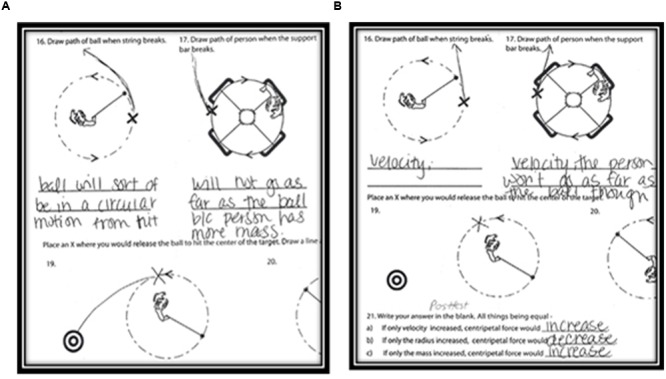
**Example of pre-test and post-test answers from same participant showing that the impetus model has been corrected**.

Items 14 through 21 were completed using a paper-based generative (off-line) subtest. The first seven items were designed to allow the student to create, with few constraints, answers to questions about several key CF concepts. The first two items displayed images of a bob swinging around a pivot (a human or a tetherball pole) and the participant was prompted to draw an arrow representing the “force on the ball,” (i.e., CF). The first five items also ended with the prompt, “Explain how you decided to draw the arrow the way you did.” These were scored 0–2. The next three items displayed bird’s eye views of CF events and queried the participant to draw arrows that represented the path that the ball (or person) would take when released from CF at a point on the circle labeled with an X. Explanations were requested as well. The final two items were near-transfer items and required the student place an X on the dotted circular path to indicate where to release the ball so that it would hit the target. The left panel in **Figure 9** shows the same participant’s answer in the pre-test, on the left and post-test, on the right. That participant held the incorrect impetus model at pre-test, but drew a correct straight trajectory for the ball after release at post-test, correcting the misconception.

#### Reliability

Two scoring techniques and reliabilities are described here: consensus tangent scoring and inter-rater correlations. For the seven off-line generative subtest items that required a tangent to be drawn (items 14 through 20). The instructions requested that the participant draw either “arrows or lines” to show the path of a released object. The veracity of the tangents was assessed by two blind scorers who needed to come to consensus. The subject ID with condition was blocked out on the top of each page. Both scorers sat together and needed to agree when there was a dispute. First, an expert overlay sheet (created by the first author) was made with the correct tangent drawn. Each participant-constructed tangent received two sub-scores. The first sub-score addressed whether the tangent appeared straight, range 0–2. The second sub-score was more objective and included the degrees of deviation from the expert angle at the circle. Theta, 𝜃, was measured with a protractor. A zero score was awarded for any angle greater than 39° from the expert-generated angle, one point for any angle between 20 and 39°, and two points for any angle less than 20° from the expert-generated angle. The two sub-scores where then summed and divided by two, thus the *mean* score of the two agreed-upon sub-scores was entered as the one score for that item (range 0–2). This was a way to take into account both global curve and degree of curve at start point; it penalized the occasional drawing that started out straight with an exact angle match at the beginning, but ended with an anomalous curved tail.

The other test items were comprised of multiple choice and short responses to open-ended prompts. A rubric for the prompt answers was created by a physics teacher and scores ranged from 0–2. Half of the tests were randomly selected for blind review. A first pass on scoring was made by a graduate student blind to condition. A second pass was made by a research assistant blind to condition on a random subset of half the tests. This research assistant was trained on the rubric and scored all items except the line drawings. Inter-rater reliability was high on the pre-test, Pearson *r* (54) = 0.98, *p* < 0.001, and acceptable on the post-test, *r* (54) = 0.81, *p* < 0.001.

### Procedure

After the pre-tests and a 3-min introductory video on vocabulary terms, the experimental intervention began and lasted approximately 50 min. The two experimenters each followed a script based on the *Tasks* subsection below in “Tasks. The Same Procedure with Eight Tasks Was Followed in Each Condition.” All lessons were advanced with a remote control held by the experimenter. Participants then took the immediate post-test. Five to six days later, they were reminded via email to return to the laboratory for the follow-up test (mode of return days for follow-up = seven). Follow-ups could occur between day 7 and 10 post-intervention. One research credit and 10 dollars were offered as further incentive to return (69 of the original 105 participants returned for follow-up).

Participants were randomly assigned to one of six conditions. The intervention or lesson included eight tasks that focused on the relationships between CF and (a) speed, (b) radius, and (c) mass – the variables in the equation. Although the tasks and learning goals were the same across the six conditions, the specific implementations varied by platform affordances and the degree of embodiment.

#### Tasks

The Same Procedure with Eight Tasks Was Followed in Each Condition.

##### Speed

The participant was instructed to vary the speed of the bob and was prompted to talk aloud about observations. There were three subtasks in this section. In the first subtask, the participant manipulated the speed of the bob (by congruent or non-congruent gestures in the high and low embodied conditions, respectively), and the speed was indicated by the dots in the green arc projection (**Figure 2**). The participants were prompted to explain why the dots in the arc trail spread out with an increase in speed, and to describe how the audio (the pitch) was affected by speed of the bob. If incorrect three times a row the experimenter supplies the answer. In the second subtask, a digital arrow perpendicular to the length of the string and tangent to the arc at bob location was added. The arrow became elongated with increased speed. The participant then varied the speed of the bob and observed changes in the length of the arrow. The participant was then asked if s/he knew the difference between velocity and speed. If the participant’s answer was incorrect on the third prompt the correct answer was supplied. (The corrective procedure was the same across all conditions, the correct answer was supplied after three incorrect attempts). Third, a purple bar graph that indicated speed was projected, and the participant was asked to match the speed of the bob to a target level shown on the purple graph. The participant was asked to explain the relationship between the speed of the bob and the bar graph. Throughout the intervention, at the end of each of the eight tasks the participant was asked to summarize how the graphical representations related to the manipulation of the bob.

##### Trajectory at release

The participant was instructed to imagine him/herself as the bob and asked to either trace (if on the IWB or desktop conditions) or walk (if in the *SMALLab high* condition) the path that would be traveled by the bob if the string were to break at a point designated by a red “X” projected onto the bob’s circular path. Participants were then asked *why* they created that path at the point of release. If the answer was incorrect, e.g., was curvilinear, s/he was prompted to try again. The participant was asked to explicitly state that objects travel in a straight path to the circle when released from circular motion. Once the idea of a straight line was established, the red “X” was moved to a different position along the circular path and the participant was asked to repeat the exercise for a total of four times predicting the traveled pathway.

##### Target Game

The participant was then advanced to the third task, the Target Game. A projected red bull’s eye target was placed in one of four locations. In the low embodied conditions, participants released the virtual bob with various methods: press a button on either the IWB pen or mouse, or use an upward “swiping” motion with the wand in *SMALLab*. In the three high embodied conditions the participants physically started the bob swinging with the input device associated with platform, e.g., in the *SMALLab* platform participants spun their bodies around in a circle and released the flinger with the brass lever; in the IWB platform participants moved the pen in a circle and then released; in the desktop platform participants spun the mouse in a circle on the table before releasing the mouse button. Thus, the high embodied participants received four trials of physical practice spinning and releasing. The low embodied participants stopped and started four simulations of the release. In all conditions, when the target was hit, audio feedback of clapping was played. The target moved location for each trial.

##### Centripetal force vector

The fourth task involved exploring the relationship between speed and CF. The participant was asked to start spinning the bob in a circle and notice that a new, yellow arrow had been added to the graphic display. Participants were encouraged to adjust the speed of the bob to see what happened to the yellow arrow. The tail of the yellow arrow was placed in the center of the bob and the arrow pointed toward the pivot running down the length of the string (tether). The arrow represented the pulling force exerted by the string (CF). The participant was then introduced to a real-time tracked yellow bar graph and asked what it represented. If incorrect a third time the answer was supplied. Alongside the graph, a target marker was placed on either the number 2, 4, 6, or 8 and the participant was asked to swing the bob so its force would match the target value. When the value was matched, the participant received audio feedback in the form of clapping and cheering. Upon completion of the two trials, the second, purple bar graph was shown below the yellow graph and labeled “meters/second (m/s).” The yellow bar graph for force was labeled in Newtons (N), the SI unit of force. The participant was asked to spin the bob at 2 m/s. A marker was placed at the corresponding value for 1 N on the force bar graph. The participant was asked to predict what would happen to CF if the speed of the bob were to double. After the guess, the participant was asked to double the speed to 4 m/s and verify the answer. Then, s/he was asked to double the speed, again, to 8 m/s. The goal was for participants to discover that force is proportional to the square of the speed rather than linearly related to speed. The participant was asked to state this relationship out loud.

##### Radius

In the fifth task added radios to the conceptual framework. The length of the string was varied to explore the relation between radius and CF. The participant was asked to predict what would happen to the force on the string if speed were kept constant and the length of the string increased. The majority responded that he force would increase if the length increased. The participant was also asked *why* a change in force might happen. The participant was then made to try the different string lengths to verify the prediction made.

In the desktop, IWB, and the *SMALLab* low embodied conditions, the length of the string was changed by clicking a button labeled “shorter” or “longer.” In the *SMALLab* high embodied condition, the original 0.5 m string was physically replaced with a 1.0 m string. As the high embodied participants swung the longer “swinger” manipulable over their heads, they often spontaneously noted how different the sensation felt and that it was “easier” to spin the longer one. Very few participants could articulate why this might be so.

##### Mini-lesson on varying radii

The sixth task consisted of a graphical and verbal explanation as to *why* the CF is greater when the radius is smaller. This was the second misconception we wanted to address. For the lesson, two concentric circles of different radii were shown on the screen (or floor for *SMALLab).* This graphic can be seen on the left in Appendix B (also called Figure 11). There was a brief explanation of what the vectors represented, and then the experimenter would click a button on the remote and the translated vectors (on the right) were projected on the floor or screen.

The vectors were translated so that the tail of the vector at time = t was aligned with the tail of the vector at time = t + 1. Thus, the translated gray vectors in the figure show the change in direction needed to keep the bob on a circular path at two successive time points. It was explained to the participant that the greater change in direction associated with the shorter radius (string) required a greater force. That is, the angular change in the “short radius” vectors’ directions required a greater pull on the string to keep the bob on the circular path at the same speed. When the experimenters talked through these graphics, the explanations often elicited comprehending “ohhhs” from participants. This was a real aha moment for many in the experiment.

##### Mass

The seventh task required the participant to vary the mass of the bob while holding the speed constant. (To help participants hold the bob’s speed constant there was both the digital representation of the bob’s speed and variable pitch sonic feedback.) First, the participants were asked to predict what would happen if speed were held constant and mass were doubled. After responding, the experimenter encouraged the participant to add more mass to the bob and see what happened to the force. At the conclusion of this task, participants were asked to describe the relation between mass and force. In the *SMALLab* high-embodied condition a packet weighing 100 g was added to the hollow bob to double its mass (there was already 100 g in the center of the bob). This allowed participants to *feel* that an increase in force was needed to keep spinning the bob at the target speed. In the other five conditions (without that manipulable bob) an increase in mass was simulated by the projected bob increasing in size with the click of a button.

##### Applying all three variables

In the eighth and final task, the participants were asked to name the three variables that affected the CF between the string and the bob (speed, radius, and mass). In a simulation, using two of the three variables, with the third variable held constant, the participants were asked to manipulate the two variables to match a target force. They practiced this until correct. After successfully matching this target force on a bar graph, the experimenter described a situation in which someone was swinging a bucket around in a circle overhead as fast as possible. The participants were asked which two factors could be changed to increase the force between the person and the bucket.

#### Experimenter Fidelity

The two experimenters moved the participants through the sections with remote clickers (*SMALLab*, IWB) or hitting the appropriate advance keys on the keyboard (desktop condition). Because the experimenters actively queried at the end of each section and also answered participants’ questions; we refer to this a “guided inquiry” lesson. Both experimenters memorized a script they helped to write. At the end of each section the experimenter would inquire about the relationship between all the elements in the task. Lessons varied only slightly between condition and almost every response from an experimenter was scripted. The experimenters clicked to advance to preordered sections after the participant answered queries correctly or the experimenter had supplied the correct answer after three attempts. There was little room for experimenter variability. The experiment lasted two semesters (∼5 months) with the same two experimenters. The first author observed each experimenter twice in the first month of the experiment. The only feedback given – and this was given to both the experimenters – was to be certain to make sure that if a participant supplied an incorrect answer three times in a row, only then should the experimenter gave the correct answer (e.g., one experimenter gave an answer after two attempts on one task, and the other experimenter gave an answer after four attempts on a task).

## Results

Invariant tests were administered at three time points: pre-test, post-test, and a delayed test. There were no significant differences on pre-tests between conditions, *F* < 2.0. A two factor factorial ANOVA that included the between-subject factors of platform and level of embodiment was used to analyze (a) the total pre-test score, (b) the on-line (declarative recall) subtest, and (c) the off-line (generative) subtest. There were no significant main effects or interactions in any of the pre-intervention analyses.

### Analyses on Whole Test

An ANOVA was run on the whole test (both subsections) analyzing the difference between pre-test to post-test (within-subjects) in addition to platform and level of embodiment. The overall immediate learning increase from pre-test to post-test was significant, *F*_(1,99)_ = 459.89, *p* < 0.001. However, all of the groups improved similarly, with no other main effects or interactions reaching significance (*F*’s < 2.0). Of the 109 students who completed the immediate post-test, 69 also completed the delayed test. The attrition rate did not differ across the six conditions, X_(5)_^2^ = 2.32, *p* = 0.80. An ANOVA was conducted using post-test to follow-up as the within-subjects variable, i.e., on delayed learning gains, and there were no significant group differences. In Appendix C are the tables for the descriptives and effects sizes for total test scores (the sum of both subtests).

### Analyses on the Subtests

Because we had reason to suspect that the high embodied groups might perform differently on the more generative subtest that were more sensitive to embodied learning, we analyzed those separately. There were no significant pre-test differences on the subtests (*F* < 2.0). **Tables 3** and **4** list the Means and SDs for the on-line (declarative) and the off-line (generative) subtests.

**Table 3:** Descriptives for on-line declarative subtest.

Condition	Pre-test *M (SD)*	Post-test *M (SD)*	Follow-up *M (SD)*
*SMALLab* low embodied	10.39 (5.63)	21.64 (4.42)	22.07 (2.89)
*SMALLab* high embodied	9.22 (4.47)	20.43 (4.83)	21.25 (3.68)
IWB low embodied	9.28 (4.45)	20.47 (3.57)	20.78 (3.46)
IWB high embodied	11.38 (4.92)	21.16 (3.86)	21.29 (4.11)
Desktop low embodied	9.69 (4.80)	20.81 (5.19)	20.18 (6.66)
Desktop high embodied	9.92 (3.13)	21.66 (2.00)	20.58 (4.50)

**Table 4:** Descriptives for off-line generative subtest.

Condition	Pre-test *M (SD)*	Post-test *M (SD)*	Follow-up *M (SD)*
*SMALLab* low embodied	16.86 (8.18)	25.86 (5.28)	25.81 (6.04)
*SMALLab* high embodied	16.48 (7.66)	24.10 (7.31)	25.60 (6.45)
IWB low embodied	13.34 (8.84)	25.12 (6.37)	21.89 (8.91)
IWB high embodied	15.44 (8.70)	26.59 (3.57)	27.25 (2.60)
Desktop low embodied	11.88 (8.95)	23.09 (6.62)	25.14 (6.44)

The two subtests varied in important ways. First, the on-line declarative subtest was taken on the computer, and its scores reflected more of the ability to retrieve knowledge rather than apply knowledge and demonstrate it in an unconstrained manner (e.g., no multiple choice items are on the generative subtest). Second, the majority of the items on the off-line generative subtest items required a type of gestural congruency to produce the answer. Participants needed to draw or generate trajectories to show the path that the bob would take when released. It was hypothesized that the congruency between learned content (more sensorimotor activity) and assessment metric might be felicitous for those who learned in a more isomorphic manner, i.e., those in the high embodied groups who practiced releasing the bob with more gestural congruency.

### Delayed Effects

**FIGURE 10:**
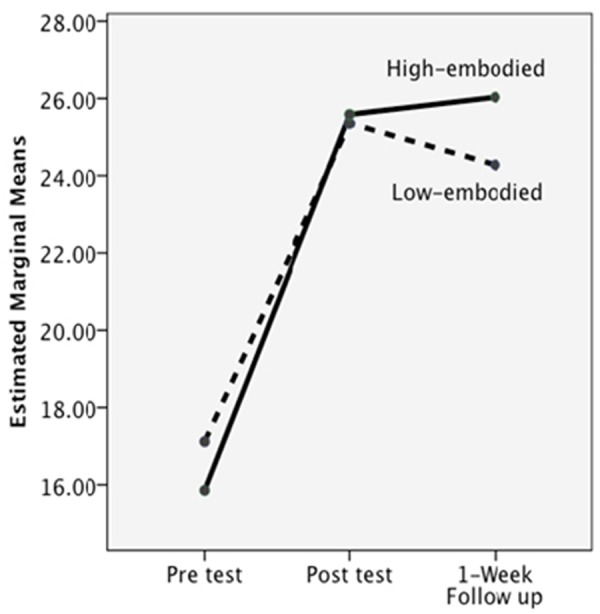
**The significant interaction from post-test to delayed on the off-line knowledge test, collapsing across platform**.

In **Table 3**, the gains between groups are almost “lock step similar.” ANOVA analyses on group differences resulted in *F*’s < 2.00, and all analyses on time resulted in *F*’s < 2.00. However, for the generative subtest, there was a significant interaction between level of embodiment and post-test to follow-up (i.e., delayed learning gains), *F*_(1,62)_ = 4.83, *p* = 0.03. As depicted in **Figure 10**, at the post-test, the low and high embodied groups performed similarly, but, with the passage of 1 week, the low embodied group did not retain as much of their new understanding of CF compared to the high embodied group. The largest decrease in retention was seen in the low embodied IWB group. The figure presents the results collapsed across platform.

### Aptitude by Treatment Effect

We also performed an analysis to determine if the interaction was modified by an aptitude by treatment effect. These analyses used multi-level modeling (MLM) for several reasons. First, the measure of aptitude, the pre-test score (which correlated significantly with number of semesters of high school physics, number of semesters of college physics, and GPA), was continuous. Second, this technique obviates concerns regarding sphericity. In addition, because this method uses maximum likelihood estimation, missing data are optimally handled. In the analyses, two degrees of freedom for platform were used to represent *SMALLab* versus the desktop and IWB versus desktop. The following variables were entered into the model and all variables were centered at the grand mean: the pre-test score, a variable representing delayed testing (delta of post-test to delayed follow-up), and the two effects-coded variables representing platform, and degree of embodiment. In addition, we entered interactions of pre-test by embodiment, pre-test by delayed, embodiment by delayed, and the three-way interaction of pre-test by delayed by embodiment. There was a significant main effect of the pre-test, *t*_(106.35)_ = 5.05, *p* < 0.001 and a significant two-way interaction of embodiment by delayed learning gains, *t*_(69.97)_ = 2.52, *p* = 0.014. Both of these replicate effects reported above. No other effects were significant (all *p*’s > 0.17), indicating no further aptitude by treatment interactions.

## Discussion

The study began with three predictions, that: (1) platform would be predictive of both immediate and delayed learning gains; (2) level of embodiment would be predictive of both immediate and delayed learning gains; and (3) the individual condition with the greatest changes would be the MR *SMALLab* high embodied one because it afforded the greatest amount of gestural congruency, sensorimotor feedback, and largest FOV. The first prediction was not supported, platform did not affect immediate learning gains. When the two levels of embodiment were collapsed, the three platform groups did not perform significantly different on post-test, nor did they perform differentially on the delayed tests. Thus, type of platform used to deliver a well-designed multimedia lesson is not a significant predictor of differential learning.

The second prediction regarding embodiment was partially supported. Although there were no differences for immediate learning, significant differences were seen on the delayed gains on the generative subtest. There was a significant effect for embodiment on the non-computerized, generative subtest, that is, from post-test to the 1 week follow-up, those who learned the content in a more embodied manner showed an advantage on recall of generative physics information. This result was primarily driven by the decrease in scores on the IWB low embodied condition and increases in scores for all high embodied conditions. This result can also be discussed using ‘levels of processing’ terminology. Those in the low embodied conditions primarily received visual and auditory feedback and used a minimum of action while learning, those in the high embodied conditions were able to use instrumented gestures with more sensorimotor feedback, while also receiving the visual and auditory feedback. Adding the motor trace is another level of processing and may strengthen the encoding signal. It may also prime the pathways that learners activated when first encountering force and it became a perceptual symbol. In addition, adding the motoric gestures may have been “disruptive” during the time of processing. Disruptive is a positive term because often being exposed to disruptive or difficult events (e.g., testing) during processing can lead to better retention and delayed learning gains (Bjork, 1994). Thus, there may be several long term advantages to including physical embodiment in the design of lessons on topics that deal with forces.

The third prediction that the *SMALLab* high embodied cell would demonstrate the greatest gains was only descriptively supported via an increase in comparative effect sizes (see Appendix C); however, inferential statistics did not reveal a statistically significant increase. The *SMALLab* high embodied condition still demonstrated the highest effect size from post-test to delayed follow-up, Cohen’s *d* = 0.22. This is non-trivial given that no physics training occurred in the interim, and it is twice the size of the next highest delayed gain (also seen in a high embodied condition).

### The Unexpected Immediate Effects

The entire group of participants displayed similar gains from pre-test to post-test regardless of condition and this was somewhat unexpected. In retrospect, there may be several reasons why this occurred. First, all six conditions were designed to engage participants and promote the development of a robust CF mental model. That is, all six lessons contained optimal inquiry-based science pedagogy (Hestenes, 1996; Megowan-Romanowicz, 2010) such as using high-quality simulations, diagrams, and active user-control of key aspects in the simulations. Even in the low embodied conditions the participants experienced more agency in navigating the pace of the simulation or animation than is offered by many of the popular science education “gizmos” (simulations or learning objects) currently available for science education. The control conditions should be considered very state-of-the-art.

From the point of view of embodiment theory, these high-quality visual simulations made all of the conditions partially embodied. That is, in all of the conditions, there were multiple opportunities to ground abstract CF concepts such as force, velocity, mass, and radius in components of the simulations. The conditions differed primarily in the amount of kinesthetics and gestural congruency. Cook and Goldin-Meadow (2006) report that “gesturing makes learning last” in the domain of learning a new mathematical concept. We saw that the condition with the most gesturing and movement via whole body (high embodied *SMALLab*) was indeed the condition in which the learning persevered more robustly.

Second, a decision was made early on that we would not consciously allow students to leave the study with incorrect mental models. That would have felt somewhat unethical. Thus, when participants answered a prompt incorrectly (e.g., replying that “a *longer* string would result in *more* CF”), participants were asked to run through the task again and to answer the question again. If they made the same incorrect conclusion three times in a row, the experimenter explicitly supplied the correct answer. This corrective guidance assured that the knowledge needed to show competency on the post-test was voiced at least one time either by the participant or experimenter. It is still worth noting that no one scored 100% on the post-test.

### Delayed Gains Seen on Appropriate Subtest

On average the participants in the three high embodied conditions demonstrated a significant delayed gain on the generative subtest. This may be due to the multiple instances of gestural congruency during encoding and because the high embodied condition elicited more sensorimotor activity. A greater amount of physical movement should activate complex motor neuron patterns and these will be associated with the learning signal. Cook and Goldin-Meadow (2006) hypothesize that their significant delay test results seen in the gesture and gesture/speech groups may be because, “…expressing information in gesture may produce stronger and more robust memory traces than expressing information in speech because of the larger motor movement.” In addition, motor planning, though unconscious, recruits resources that have downstream effects on attention and may affect delayed learning gains. We have seen similar delayed results on nutrition knowledge tests when comparing low and high embodied learning conditions in an exergame, i.e., greater retention effects were seen in the delayed knowledge tests for the active, more embodied group (Johnson-Glenberg et al., 2014b). In this current study, the generative subtest was composed of several items that required participants create answers with movements that either mimicked the movement of the bob in flight, or were generated from recall and not recognition. The act of drawing may have also gesturally reified the meaning of the encoded content.

#### Gains in the *SMALLab* High Embodied Cell

Although the greatest delayed learning gains were seen in the *SMALLab* high embodied cell, the difference was not statistically significant. There are some power concerns with the analyses due to delayed test attrition, but we will also mention two issues exclusive to *SMALLab*: novelty and technology problems. First, the novelty of the immersive *SMALLab* experience can distract from learning at first. When *SMALLab* is used in schools, participants have several days to acclimate to the technology and use the motion tracking wands. They are also able to observe peers perform. In contrast, during the experiment, there was no extended formal period of adaptation. Participants walk immediately into a very techy-looking truss system with multiple draping wires and are simply told, “Above are infrared cameras that track the motion of certain handheld objects.” In this individual experiment experience, the participant is active from start to finish with no chance to observe. Yes, it is novel and that can be engaging (see a sample dialog from a participant in Appendix D to get a sense of the flow and how engaging the platform can be). But, *SMALLab* also requires a tremendous amount of sensory integration in a very short time span when used in a non-collaborative, one shot experimental situation. In addition, there were the requisite technology woes. All 12 infrared cameras must be tightly calibrated and synced for the system to work correctly, otherwise jitter is introduced into the floor projections. The experimenters reported that four sessions seemed to have arrows that “jumped around” a bit. Consequently, error variability was introduced into some of the high embodied *SMALLab* sessions that was never present in the other five conditions. Nonetheless, the effect size in the *SMALLab* high embodied condition was twice that seen in the next highest delayed learning gains condition. This lends some support to the theory that grosser body movements and the ability to directly manipulate and feel CF effects may contribute to greater delayed learning gains. For example, in the *SMALLab* high embodied condition they were able to insert weighted mass packets into the “swinger” bob, and then swing the heavier bob overhead and directly experience greater exertion in their core. This experiential “feeling” of the effects of greater mass is probably very clarifying. It may be more effective than merely showing the bob getting larger in a graphical manner (as was done in the five other swinger-less conditions). In sum, there were both negative and positive consequences associated with the *SMALLab* high embodied condition.

### Creating Embodied Content

It may be the case that certain topics may lend themselves more readily to being taught in an embodied manner. Perhaps it is more straightforward to teach about forces with the body than to teach about justice. We can assert with some assurance that the amount of embodiment in a lesson is important for delayed learning gains in the context of this CF experiment. Although we were surprised that platform did not have an effect, it may also be the case that being freed from platform dependency is a very good thing for education. Creating an optimal lesson might not be highly dependent on the exact technology used. Optimal lessons, those that encourage retention of knowledge, may rely more on the extent of gestural congruency and/or sense of immersion designed into the lesson and less on the technology. A recent example of an embodied lesson delivered via a simple “instruction animation” on a computer monitor comes from Pouw et al. (2016). The topic was levers and a seesaw analogy was provided to the middle school students. They were encouraged to stretch their arms out and think of their torsos as the fulcrum. In the study virtual weights appear on an avatar’s outstretched arms and the students must decide which weight is heavier. This is a succinct and elegant meshing of body metaphor with applied science, and it does not require expensive technology to deliver the message.

If it is the *amount of embodiment designed into the lesson* that is crucial, and teachers do not necessarily need large truss systems, etc., to activate embodiment, then by creatively designing for mouse and extant tracking pads, we should be able to produce highly embodied content. Instructional designers should strive to create lessons that are highly embodied, generative, and include gestural congruencies that are well-mapped to the learning goals. In addition, new cost-effective motion tracking technologies are rapidly entering the market, including VR units that track and respond to hand position. We are optimistic these technologies will come down in price and make their way into classrooms. When that happens, we stress that teachers needed to be properly trained to use new media techniques. Nathan and Alibali (Nathan et al., 2014) found a relationship between action and cognition and experimenter’s language (prompts and hints) as participants learned geometry proofs. This suggests that providing guided scripts for instructors is important to get the most out of a technology-supported, mediated embodied lesson.

One way to think about the undifferentiated immediate gains in this study is to note that the “instructors” were highly trained (the experimenters actually helped to write the physics script and were experts). The participants learned via one-on-one guidance with the correct answer eventually supplied, thus, we may have created an artificial plateau for gains. Perhaps the novice learners could learn no more due to the platforms’ influence because the human instruction quality was so high? The constructs to be varied in a future study could deal with levels of embodiment as well as quality of the instructor. Personally, we are interested in designing technology to aid teachers, not supplant them. We do not always insert guiding avatars into our systems. Thus, we highly recommend that designers focus on professional development and lesson plans as well. Interestingly, Harris and Sass (2007) found that teacher pre-service training generally had little influence on student productivity. One exception was that content-focused teacher professional development was positively associated with productivity in middle and high school math though. Designers of mediated STEM lessons need to also take time to create lesson plans for teachers. Not only plans, but teachers should receive specific training on mediated, embodied lessons multiple times immediately *preceding* use of the specific technology. We delve more into professional development.

### How Does the Embodiment Taxonomy Relate to These Physics Lessons?

Finally, the field needs to use the term “embodied” in a more codified manner. Which is why we continue to refine the Educational Embodiment Taxonomy (Johnson-Glenberg et al., 2014a). We have left an explication of the six lessons to the end, should the readers wish to make their own mappings before we put forth ours. Again the three constructs were: (a) amount of sensorimotoric engagement, (b) gestural congruency, and (c) immersion. Using the taxonomy, we suggest the six lessons be classified thusly:

4th degree – *SMALLab* high embodied.

3rd degree – Both *SMALLab* low embodied and IWB high embodied conditions because many of the gestures were congruent (although these conditions were not as haptic and kinetic as *SMALLab* high embodied), and both contained larger FOVs.

2nd Degree – Both IWB low embodied and desktop high embodied conditions because the first had a larger FOV, and the second allowed for gesturally congruency – albeit via smaller circular mouse movements.

1st degree – Desktop low embodied because the learner primarily stopped and started the simulations so there was no gestural congruency, and it contained a smaller FOV.

What would a truly non-embodied lesson look like? Such a lesson might be text only, it would never makes reference to anthropomorphization (“flip your hand to transpose a table”). The non-embodied text could include symbols, but no pictures (images), no animations, nor auditory cues (e.g., pitch increase with speed). It would not be multimodal. On the other hand, there is no guarantee that a learner would not spontaneously create a rich visual mental model from text alone; if perceptual symbols are unconsciously activated, then can any content ever be truly “unembodied”?

### Future Directions

This is one of the first attempts to use highly embodied methods to teach physics in a rich multimedia MR environment. Important lessons in design for content with congruent gestures were learned. The ultimate goal is to design lessons for classrooms, to make sure the best pedagogies have a broad reach. We were also able to pilot the CF lesson in an 11th grade science classroom with *SMALLab*. We have observed *SMALLab* in this high school setting for several years. We have consistently seen that interest from the observing students (only 1–4 students can be active in the center at one time) is always high the first half of a class session, but attention begins to wane after (a) a student has been active, or (b) they have observed up to four rounds. One way to keep the observing students engaged is to assign tasks to small groups (a strong technique borrowed from Reciprocal Teaching by Palincsar and Brown (1984) and Rosenshine and Meister (1994). Groups of three to four students ring the space around *SMALLab* and each group holds a small whiteboard. The groups write down their predictions, for example, if speed is increased will CF increase linearly? Yes or no, and why. Each round would be evaluated post-performance and the prediction shared with the entire class; discussion was encouraged as to why a prediction was correct or incorrect. This sort of student-centered teaching with technology may not be something all teachers are familiar with.

It is our job as designers to think through how the content will be used in real classrooms and to also guide teachers in how to be most effective. We encourage designers of educational technology to think through the non-tech parts of the lesson as well. Lesson plans should be available and within each lesson designers need to build in time for student reflection and discourse. There have been instances where we allowed teachers to use our embodied systems after only one webinar worth of training, in general those have resulted in less successful lessons. We give space to the topic of professional development here because even if a designer creates a seamless lesson that sublimely meshes technology and embodiment, when a teacher does not know how to use it, it is wasted experience for all. Teachers’ practices are crucial, and many teachers are interested in integrating more technology into their lessons. Recent research supports that subject areas are key predictors in the success of technology integration; however, the effect of subject area on technology integration is not well understood (Howard et al., 2015), however, it seems in the area of STEM technology is more readily integrated. The model for professional development-at-a-distance is changing and we need to proactively support teachers who are use cutting edge technologies with multiple training sessions and access to videotaped real world lessons.

We predict that results from this study will generalize beyond CF. Indeed, the team has created embodied content for, and researched in, a range of topics including: gears (Johnson-Glenberg et al., 2015), disease transmission (Johnson-Glenberg et al., 2014a), geology (Birchfield and Johnson-Glenberg, 2010), and metaphor comprehension using other motion capture techniques and *SMALLab* (Hatton et al., 2010). The MR sessions that have been most successful are the ones where the teachers had very active roles in co-designing the content and/or they received more than two sessions of on-line training.

More research is clearly needed on emerging learning technologies and how various technologies can afford more or less embodiment. AR with tablets or smart devices is poised for large scale dissemination in classrooms. Designers need to be creative about adding gestures to learning with small form devices and mapping gestural congruency. The accelerometer should be integrated into lessons where add value is predicted (e.g., force in physics). Little is known about how the observing students are affected by learning in highly mediated high embodiment lessons (although see Kontra et al., 2015). Less is known about how gestures affect a learner’s sense of agency or further enjoyment and engagement while learning. We are encouraged by those who create immersive classrooms (Lui et al., 2014) and use MR platforms in educational and informal learning spaces (Lindgren and Moshell, 2011; Tscholl and Lindgren, 2014) and the gains reported in learning. We do not see technology receding from the modern classroom.

### Lessons for Designers

One foremost tenet for designers is to design for interactivity. The learner should be able to *create* content as s/he learns. Learners also need immediate, yet non-disruptive, feedback. In the field of STEM, how many high-quality, *interactive* science objects are available for free? The PHET simulations are outstanding examples, but there is currently not one for CF. The first 25 objects in a Google search on CF (date of retrieval September 2016) were observational videos or worksheets. Not one hit in the first few pages was an interactive multimedia object. We should fill this void (a void in STEM overall) by creating browser-enabled objects that encourage learners to use a mouse or tablet surface as a gesture-based interaction tool. For example, for CF, the learners should be able to spin a bob and release it with user-generated actions. There should be multiple chances for exploration and failure as the learners try to hit a stable target. Learners should be able to draw predictive paths and vectors and then assess the veracity of their actions. They should spin and release a bob and try to hit stable, then moving targets as they “leveled up” and reify concepts. The action should match the learning goal, e.g., if speed of spin is important, then the speed at which the finger circles on the screen needs to part of the displayed information. That is a strong example of gestural mapping and congruency. A weaker example would be a lesson where the learner types in a number for speed and the bob automatically spins to a facsimile of that speed. That is not gesturally congruent. When new media instructional designers understand the limits of the technology and how to map gestures to the key content to be learned (e.g., don’t use a hand “push” motion with a *Kinect* to set a gear train spinning), use a hand “spin” motion, (Johnson-Glenberg et al., 2015), then larger and more sustained learning gains may be seen.

Creating quality educational content also encompasses the concepts of ecological validity and transferability. This is one of the problems pointed out by Shute et al.’s (2015) analysis of a well-designed videogame compared to a popular computerized brain training regime. What is being taught should promote generalization and learning transfer beyond the “actual tasks” performed in the intervention. Although we believe that embodiment principles *generalize* to other topics, we cannot say if the specific content learned in our lesson will *transfer*. This current study focused on delayed gains and did not assess transfer to other far domains (e.g., how is the *r* in the CF equation denominator similar to the *r* in the equation for Coulomb’s law?). We hypothesize that when transfer does occur it may be because that lesson created a situation where the learner’s p-prims no longer contain misconceptions *and* the knowledge pieces have been well-integrated into the previous knowledge structure so the new pieces can now be applied to other scientific constructs. In this study, the majority of participants who once held the impetus model had corrected it by post-test.

#### New Assessments

New types of assessments should be included with new interactive learning objects. Not only does this satisfy some constraints associated with encoding specificity (i.e., Tulving and Thomson, 1973), but the field should continue to research whether knowledge is indeed retained longer when learned in a more embodied manner. Testing is itself a method for increasing learning (Roedinger and Karpicke, 2006) and new embodied methods for testing should continue to be developed. It is now easy to gather in-process data while the participants are in the act of learning (Plass et al., 2013) and creative assessments should also take advantage of what motion capture data can reveal. Affordable new technologies (e.g., Microsoft *Kinect*, Intel *RealSense*) provide rapid sampling of rich data streams and reveal how learners move through 3D space and make decisions during learning. The trick is to design the content to elicit meaningful gestural actions at key decision points during the lesson. Recently our lab ran a study using randomized control trials on the topic of electric fields and vectors. In a traditional post-intervention declarative style test, the participants in the high embodied groups did not show a difference in learning compared to low embodied. However, when post-intervention knowledge was assessed with a large Intuous *Wacom* tablet that afforded and measured acceleration of hand-drawn vectors, then the high embodied conditions demonstrated significant knowledge differences (Johnson-Glenberg and Megowan-Romanowicz, submitted). Instrument sensitivity may play a role in this current study as well, it appears that allowing participants to generate and draw freehand on paper (the off-line subtest) revealed knowledge that could not be gathered on the less sensitive keyboard-based task (the on-line, declarative subtest).

#### Sleep and Consolidation

Finally, the delayed test results raise issues regarding sensorimotor, gestural signals, and delayed effects. What is driving the delayed effect, and can it be enhanced with more or less delay? Memory consolidation is surely occurring; skill acquisition and procedural knowledge are known to be affected by sleep (Walker and Stickgold, 2004; Stickgold and Walker, 2007), but little is known about how type of learning (embodied or not) interacts with optimal length of sleep for memory consolidation in humans. Multiple follow-up test points may address this.

## Conclusion

The experiment examined the effects of embodiment and learning platform on immediate and delayed learning. All participants made impressive significant gains on the immediate post-test. Prior to data collection, we hypothesized that students in the highly embodied conditions would outperform those in the low embodied conditions. We did not see this result at immediate post-test; however, we did see significant gains for the high embodied group on the 1 week delayed subtest that allowed the participants to be more generative. Participants retained more generative physics knowledge after learning in the highly embodied lessons regardless of the learning platform. This may be because adding an extra motor trace during encoding of new knowledge strengthens or “coheres” new knowledge to old knowledge structures in a more felicitous manner.

Based on these results, two general conclusions are drawn. First, principles of embodiment can be applied effectively for the enhanced delayed learning gains of certain STEM material and for overcoming incorrect mental models. Second, we suggest that instructional designers create lessons that contain more embodiment when possible, this means considering the amount of sensorimotor engagement, gestural congruency, and immersion when designing. It appears that the effects of high embodiment may be more robustly revealed on delayed tests. Designers who only have access to mouse and keyboard technology can still be creative about how to make the content gesturally congruent, e.g., spinning the mouse in a circle for a CF lesson. Given that education is about retaining material for future application, it is important that assessment measures be given several times after an intervention. The assessment measures should be sensitive to the instructional methodologies, e.g., including hand-drawn objects may reveal differences in learning. Finally, we are hopeful that the emergence of cost-effective forms of motion tracking technology herald a new age for the inclusion of more embodied content into classrooms, and that teachers will be more effectively trained in how to use these new methodologies.

## Author Contributions

MJ-G co-designed project, oversaw study, analyzed results, and wrote majority of the manuscript. CM-R co-designed study, acted as physics expert, created tests, wrote much of the introduction. DB co-designed study, coded some of the content, co-created the mixed reality platform called SMALLab. He then left ASU and had no input in the data analyses, results, or discussion sections. He is an unbiased co-author. CS-R was the lead experimenter as a graduate student and helped to co-design the tests.

## Conflict of Interest Statement

DB is listed as a co-author because he helped to write the NSF grant that secured the funding for the project and he contributed in the first year to co-design the study and program some of the conditions. In year two of the grant when participants were recruited, Dr. Birchfield decided to leave academia and the PI-ship was handed over to the first author. DB moved to California to run the company called SMALLab Learning, LLC. www.smallableanring.com. The site is dedicated to embodied learning, but there is no content for sale on that site that corresponds to this work or the topic of centripetal force. The scenario described in this manuscript is not a commercial product. DB had no input into the project after the creation of the six conditions. He never worked with the data nor wrote sections in this article. Nonetheless, he deserves co-authorship because it would not have come to pass without him. All the other authors declare that the research was conducted in the absence of any commercial or financial relationships that could be construed as a potential conflict of interest.

The reviewer CL and handling Editor declared their shared affiliation, and the handling Editor states that the process nevertheless met the standards of a fair and objective review.
