# Supplementary material for: Effects of Embodied Learning and Digital Platform on the Retention of Physics Content: Centripetal Force
Source: Front Psychol. 2016 Nov 25;7:1819. doi: 10.3389/fpsyg.2016.01819 (PMC5122822; doi:10.3389/fpsyg.2016.01819)
Supplement: Supplementary file 1 [file Image_1.pdf]

frontiers

Supplementary Material

A Taxonomy for Embodiment in Education and a Mixed Reality Physics Study Supporting Delayed Embodied Learning Effects

Mina C. Johnson-Glenberg 1 *
Colleen Megowan-Romanowicz 2
David A. Birchfield 3
Caroline Savio-Ramos 4

1 Department of Psychology, Arizona State University, Tempe, AZ, USA and Behavioural Sciences Institute, Radboud University, Nijmegen, NL
2 American Modeling Teachers Association, Sacramento, CA, USA
3 *SMALLab Learning*, LLC, North Hollywood, CA, USA,
4 Mary Lou Fulton Teachers College, Arizona State University, Tempe, AZ, USA

*** Correspondence:**
Mina C. Johnson-Glenberg
Mina.Johnson@asu.edu

Supplementary Appendices:

Appendix A – Pages 1 and 2 are the online test questions; items 14 through 20 represent the more generative test items.

Centripetal Force Test - Online Version, p. 1 of 2

1. Define "force".

2. What is the name of the force that causes an object to travel in a circular path?

3. List all the factors you can think of that affect an object traveling in circular motion.

4. Define "velocity".

5. Consider a tether ball connected by a rope to a pole. What is the force that causes the ball to travel in a circular path around the pole (the correct answer is NOT circular force)?

6. Carol and Jack are holding hands and Carol is spinning Jack around in a circle. Describe all Jack's sensations as he is being spun around.

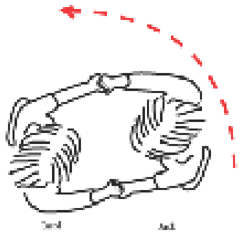

FIG. for Question 6: Carol and Jack spin in a circle.

7. Consider a tether ball system like the one shown to the right in which the rope is perpendicular to the pole as the ball travels along a circular path around the pole. If the velocity of the ball is doubled, the force needed to keep the ball in the same circular path will:

a) Remain constant
b) Double
c) Be cut in half
d) Quadruple

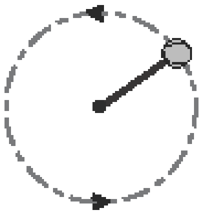

FIG. for Question 7: Tether ball swinging around a pole.

8. Examine the pictures to the right. Which system involves the greatest centripetal force?

a) image A - 1 meter per second
b) image B - 2 meters per second
c) image C - 3 meters per second
d) All conditions equal

Explain why you chose your answer.

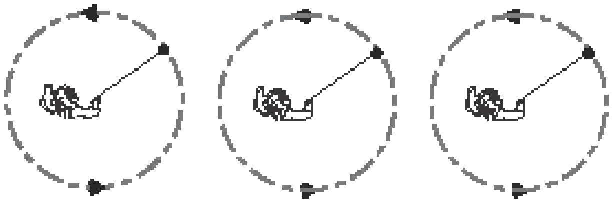

1 meter / second image A | 2 meters / second image B | 3 meters / second image C

FIG. for Question 8: Person swinging ball around at three different speeds.

9. Which of these people is experiencing the greatest centripetal force?

a) The person on the larger merry go round
b) The person on the smaller merry go round
c) The people experience the same amount of centripetal force
d) Neither person is experiencing centripetal force

Explain why you chose your answer.

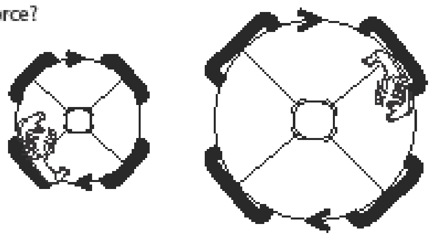

FIG. for Question 9: Two identical twins are on two different merry-go-rounds. The left merry-go-round is smaller; the one on the right is larger. Both twins have the same mass, and each are traveling at 2 meters per second (e.g., 2 meters/sec).

10. Imagine a person is swinging the ball around in a circle. The person swings the same ball around at the same speed three times, each time using a rope of a different length. In which of these situations is centripetal force greatest?

a) image A - shorter
b) image B - longer
c) image C - longest
d) All experience the same amount of force

Explain why you chose your answer.

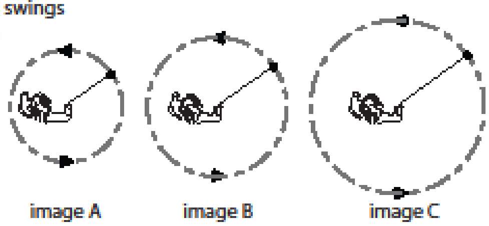

FIG. for Question 10.

11. Consider a tether ball system in which the ball is traveling at a constant 2 meters per second. Now imagine that the length of the rope is doubled. The force needed to keep the ball traveling in its circular path at the same speed as before will:

a) Remain constant
b) Double
c) Be cut in half
d) Quadruple

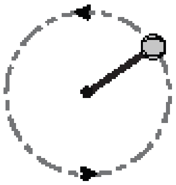
FIG. for Question 11: Tether ball swinging around a pole.

12. Now suppose the mass of the ball is doubled. The force needed to keep the ball traveling at the same speed (2 meters per second) in the same circular path will:

a) Remain constant
b) Double
c) Be cut in half
d) Quadruple

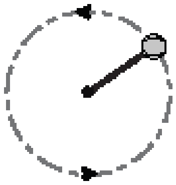
FIG. for Question 12: Tether ball swinging around a pole.

13. In the system pictured to the right, the larger the ball, the greater the mass. Which of the three situations pictured entails the greatest centripetal force?

a) image A - smaller mass
b) image B - larger mass
c) image C - largest mass
d) All systems equal

Explain why you chose your answer.

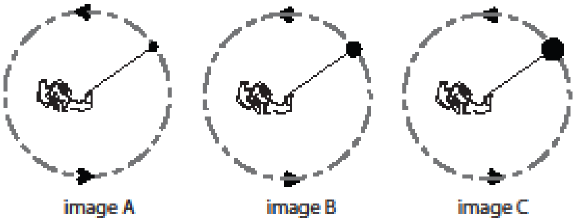

FIG. for Question 13.

Circular Motion Assessment Pre-test

ID: ____________________

Ignore the effects of gravity in the following items.
For each of questions 14-18, explain how you decided to draw the arrow representation the way you did.
Write your answer on the line beneath each question.

For the next two questions, draw an arrow to represent the force on the ball.

14. Person swings the ball over the head.

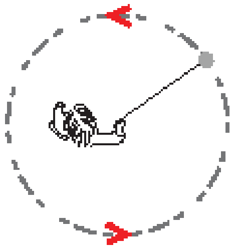

15. Tether ball rotating around a pole.

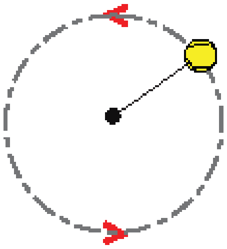

For the next three questions, draw an arrow to show the path that either the ball or person would take when released at point X. Please ignore the effect of gravity; we care about the horizontal direction that the ball or person travels.

16. Draw path of ball when string breaks.

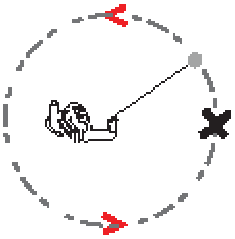

17. Draw path of person when the support bar breaks.

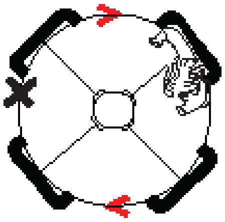

18. Draw path of ball when rope breaks.

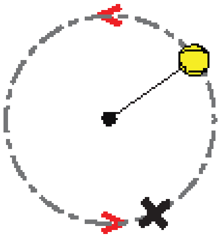

Place an X where you would release the ball to hit the center of the target. Draw a line as well to show the path the ball will take.

19.

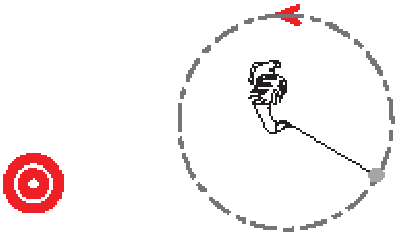

20.

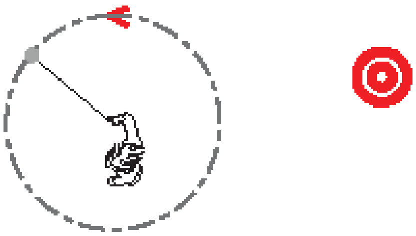

Appendix B. The graphic displayed for radius section

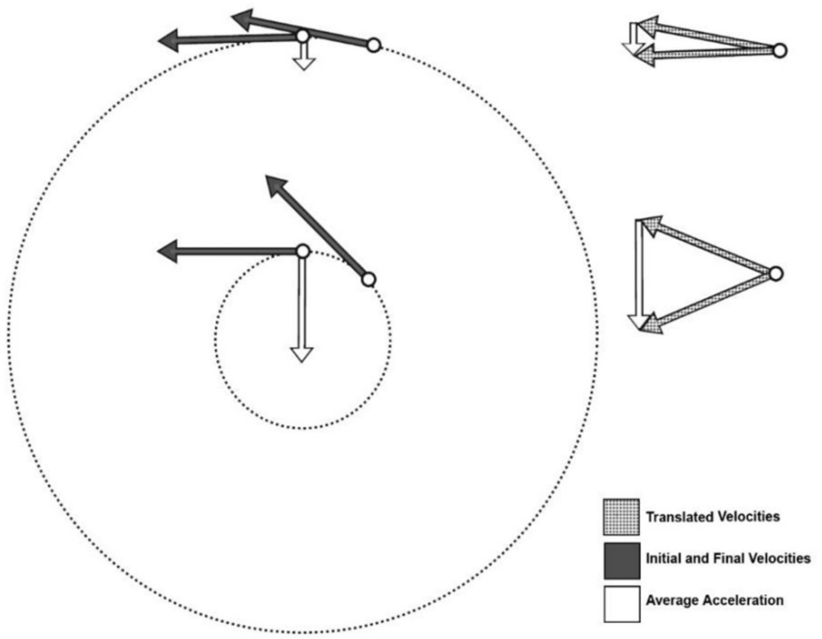

Figure 11. Screenshot of the graphic used to explain why more CF is needed when a circle is smaller (i.e., the radius is shortened) and the same speed is maintained.

Appendix C. Table C1. Total Test Scores with Effect Sizes

Condition	Pretest Mean (SD)	Posttest Mean (SD)	ES Pre to Post	Followup Mean (SD)	ES Post to Followup
SMALLab Low Embodied	28.13 (11.86)	47.50 (8.29)	1.69	47.29 (7.50)[1]	-.02
***SMALLab* High Embodied**	**26.02 (11.15)**	**44.52 (10.59)**	**2.41**	**47.07 (9.50)**	**.22**
IWB Low Embodied	22.62 (12.10)	45.59 (8.79)	2.12	42.67 (11.83)	-.06
IWB High Embodied	26.82 (10.09)	47.75 (5.56)	2.53	48.43 (6.57)	.08
Desktop Low Embodied	21.56 (11.04)	43.91 (9.65)	2.22	45.05 (11.72)	.10
Desktop High Embodied	22.75 (10.54)	46.63 (6.10)	2.01	45.83 (5.80)	-.01

[1] n = only 12

Appendix D

Sample dialogue in SMALLab high embodied condition. Below is a learning-in-action sample dialogue from a female participant in the *SMALLab* high embodied condition. She was unusual in that she reported having two semesters of high school physics. The participant (P) is taken to the center of SMALLab floor projection by the tester (T) and given the “swinger” to spin over her head.

Tester (T): “As you’re spinning that overhead you’re going to get some feedback. You notice the path on the floor, and you notice the light green dots on the path.”

Participant (P): “Yep.”

T: “Spin it a little faster and see what happens to the distance between those dots.”

P: “It gets bigger.”

T: “Good. And, as you slow down what happens?”

Participant slows down her wrist spin and observes the floor.

P: “The dots get closer.”

T: “Good, so what do you think those green dots might represent?”

P: “Um.” Pause “They indicate your speed? I guess they are taken at constant time intervals, so indicating how far the ball has traveled.”

T: “Right, so - indicating position.”

P: “Right.”

T: “Yeah, you notice the sound, too? What happens to the pitch of the sound as you swing it faster?”

P: “It grows higher.”

T: “Good, and as it slows down, it...?”

She slows her swinging.

P: “Lowers.”

T: “Right.” (*Tester pushes button on a remote she holds. This advances lesson and adds a digital arrow on top of the digitally projected bob on the ground*): “Now I am adding a purple arrow as you spin.”

(P): “Cool!”

T: “What does it mean?”

Participant begins swinging the ball overhead again.

P: “I think, well, I don’t know what it’s called, but if the string breaks it’s the direction the ball would fly off in.” <NOTE: this question was on the pretest.>

T: “Well. Yes, that, but also spin it faster and see what happens.”

P: “Oh, the arrow is getting longer.”

T: “Now spin the bob slower, what do you think that arrow is representing?”

P: “Now it’s getting shorter.”

T: “So, besides direction the arrow also represents...?”

P: “Oh, yeah, also speed, well, velocity.”

T (*clicks the remote again*): “Great! Ok ,now look down here. I have added a bar graph, what do you think that corresponds to?”

P: “Well, it’s... I dunno, speed, but is it the speed of just the ball?” (*She gets a little wrapped up in the swinger string here as it slows over her head. Untangles self. Spins bob again.*) “Let me see. Would it correspond to the length of the purple arrow?”

T: “Yes.” (*Laughs)* “Well, actually I should let you discover that yourself, just spin it and see.”

P (*spinning bob overhead*): “Yeah, so yes, it definitely does.”

T: “Good, now try to spin it at the speed marked in the bar graph, that is at eight.”

(Participant spins faster.)

T: “Good, that’s a match.”

T: “Just to recap. So how do all of these representations relate to each other? The dots, the arrow, the bar graph, the pitch - how are they all related?”

P: “They all give me speed...so, the distance of the dots, the length of the arrow, where I fall on the bar graph, and the pitch if, its high or low.” (*She swings bob again overhead a few more times.)*

“Hey, this is really fun.”
